# The scheme, and regulative mechanism of pyroptosis, ferroptosis, and necroptosis in radiation injury

**DOI:** 10.7150/ijbs.91112

**Published:** 2024-03-03

**Authors:** Jiaoyang Ning, Liu Chen, Yu Zeng, Gang Xiao, Wentao Tian, Qi Wu, Jiyuan Tang, Shuangshuang He, Guilong Tanzhu, Rongrong Zhou

**Affiliations:** 1 Department of Oncology, Xiangya Hospital, Central South University, Changsha, 410008, China.; 2 Changsha Stomatological Hospital, Hunan University of Traditional Chinese Medicine, Changsha, China.; 3 The Third Affiliated Hospital of Guangzhou Medical University, Guangzhou, China.; 4 Department of Orthopedics, Shanghai Key Laboratory for Prevention and Treatment of Bone and Joint Diseases, Shanghai Institute of Traumatology and Orthopedics, Ruijin Hospital, Shanghai Jiaotong University School of Medicine, 197 Ruijin 2nd Road, Shanghai, 200025, China.; 5 Department of Radiation Oncology and Department of Head and Neck Oncology, Cancer Center, West China Hospital, Sichuan University, Chengdu, Sichuan, 610041, China.; 6 Xiangya Lung Cancer Center, Xiangya Hospital, Central South University, Changsha, 410008, China.; 7 National Clinical Research Center for Geriatric Disorders, Xiangya Hospital, Central South University, Changsha, Hunan Province, 410008, China.

**Keywords:** Pyroptosis, Ferroptosis, Necroptosis, Radiation injury, Regulative mechanism

## Abstract

Radiotherapy (RT) stands as the primary treatment for tumors, but it inevitably causes damage to normal cells. Consequently, radiation injury is a crucial consideration for radiation oncologists during therapy planning. Cell death including apoptosis, autophagy, pyroptosis, ferroptosis, and necroptosis play significant roles in tumor treatment. While previous studies elucidated the induction of apoptosis and autophagy by ionizing radiation (IR), recent attention has shifted to pyroptosis, ferroptosis, and necroptosis, revealing their effects induced by IR. This review aims to summarize the strategies employed by IR, either alone or in combination therapy, to induce pyroptosis, ferroptosis, and necroptosis in radiation injury. Furthermore, we explore their effects and molecular pathways, shedding light on their roles in radiation injury. Finally, we summarize the regulative agents for these three types of cell death and their mechanisms. In summary, optimizing radiation dose, dose rate, and combined treatment plans to minimize radiation damage and enhance the killing effect of RT is a key focus.

## Introduction

Annually, approximately 470,000 patients undergo radiotherapy (RT) in the United States, with up to half of all cancer patients worldwide anticipated to receive this treatment 1. When integrated with surgery 2, chemotherapy 3, targeted therapy 4, immunotherapy 5, and other modalities, RT proves effective in inhibiting tumor progression, extending patient survival, and enhancing the quality of life. Mechanistically, the traditional perspective suggests that RT directly affects biological macromolecules such as DNA and proteins 6. It also indirectly impacts water molecules, generating radiolysis products that cause DNA damage and protein denaturation, ultimately resulting in tumor cell death 7. Furthermore, with the advent of immunotherapy, the role of the immune system in tumor treatment has gained attention. Numerous studies have demonstrated that RT functions as an "in situ vaccine", activating the immune system and promoting anti-tumor immunity 8.

While RT effectively eliminates tumors, minimizing damage to normal tissues remains challenging. Complications such as radiation pneumonitis, pulmonary fibrosis 9, radiation myocarditis 10, radiation brain damage 11, and intestinal damage 12 require resolution. Current clinical treatment measures for radiation injuries primarily involve symptomatic treatment 13. The combination of drugs for preventive measures to reduce radiation injury remains relatively rare 14. Radiation oncologists are dedicated to eradicating tumors while minimizing side effects on normal tissues. To mitigate radiation injury, various approaches are being explored. Radioprotective drugs like memantine 15, vitamins 16, amifostine 17, 18 (NCT00040365), melatonin 19, 20 (NCT03716583), evosk 21, and trixiera 21, 22 (NCT02334345) are explored. Advances in imaging and positioning techniques 23 provide a foundation for accurate RT. FLASH-RT, utilizing ultra-high dose rates (> 40Gy/s), has gained significant attention 24. FLASH-RT maintains local tumor control while reducing normal tissue toxicity compared with conventional RT 25-29. Furthermore, the application of particle beams in proton and heavy ion RT interests radiologists due to their potential to minimize normal tissue damage 30, 31. Despite the technological efforts, minimizing radiation injury remains a challenge 32. Continued research and innovation are necessary to further improve the balance between tumor control and normal tissue preservation in RT 13.

Cell death can be categorized into non-programmed cell death, and programmed cell death 33, 34. While apoptosis has traditionally been regarded as the primary mechanism of cell death, the recognition of alternative forms, such as programmed necrosis, has challenged this conventional perspective 35, 36. Pyroptosis 37, 38, ferroptosis 39, 40, and necroptosis 41, 42, which have gained significant attention in recent years, fall under the category of programmed cell necrosis.

Pyroptosis was initially proposed by Cookson and Brennan in 2001 43. In 2017, Shao's team revealed the role of the gasdermin family in forming membrane pores during pyroptosis 38, heightening interest in pyroptosis research. Pyroptosis is involved in various fields, including infectious diseases 44, neurological diseases 45, cardiovascular diseases 46, drug cardiotoxicity 47, and tumors 48. Introduced by Dr. Dixon in 2012 49, ferroptosis is characterized by iron-dependent lipid peroxidation and reactive oxygen species (ROS) accumulation. Ferroptosis is implicated in neurodegenerative diseases 50, drug-induced pulmonary fibrosis 51, acute kidney injury 52, cardiovascular diseases 53, and tumors 54. Necroptosis, proposed in 2005 55, represents a regulated form of necrosis influencing diseases like ischemia-reperfusion injury 56, and liver fibrosis 57, diabetic heart injury 58, Alzheimer's disease 59, and tumors 60. These programmed cell death mechanisms, including pyroptosis, ferroptosis, and necroptosis, have emerged as pivotal areas of research owing to their involvement in various diseases and pathological conditions. Understanding their mechanisms offer valuable insights for developing targeted therapeutic strategies.

Indeed, pyroptosis, ferroptosis, and necroptosis are distinct programmed cell death mechanisms, and the interconnections and transformations between these pathways remain incompletely understood. Each mode of cell death is governed by specific signaling pathways. Pyroptosis relies on caspase family and inflammasome activation, involving both caspase-1-dependent and caspase-1-independent pathways 61. Upon stimulation by signals from bacteria, viruses, and other sources, caspase-1 is activated and cleaves gasdermin D (GSDMD), leading to the release of interleukin-1β (IL-1β) and interleukin-18 (IL-18) 62. In the caspase-1-independent pathway, activation of caspases-4, 5, or 11 triggers GSDMD cleavage, resulting in membrane pore formation and the release of inflammatory factors 63. Ferroptosis is characterized by lipid peroxidation accumulation 64. The classic mechanism involves extracellular signals inhibiting glutathione peroxidase (GPXs) 65 and cystine/glutamate transport receptor (system Xc^-^) 66. This leads to the activation of p53 gene 67, voltage-dependent anion channels (VDACs) 68, and other mechanisms inducing ferroptosis. Unlike apoptosis and pyroptosis, necroptosis does not depend on caspase activity but is primarily regulated by receptor-interacting kinase-3 (RIPK3)-mediated mixed lineage kinase domain-like (MLKL) phosphorylation 69.

Ongoing research on cell death induced by IR, leading to either tumor cell elimination or normal tissue damage 32. The cell death induced by IR extends beyond apoptosis 70 and autophagy 71. It exerts its effects by triggering a variety of cell death pathways, including pyroptosis, ferroptosis, and necroptosis. Recently, radiobiologists have initiated investigation into the roles of radiation-induced pyroptosis, ferroptosis, and necroptosis. This review aims to understand the protocols and molecular regulatory pathways involved in radiation damage mediated by pyroptosis, ferroptosis, and necroptosis, both in the context of RT alone or in combination with other treatments.

### Radiation-induced pyroptosis/ferroptosis/necroptosis and dosimetry

Pyroptosis is a significant contributor to radiation injury. Studies have confirmed that ionizing radiation (IR) induces pyroptosis along with various radiation-induced injuries such as sepsis 72, radiation pneumonitis, and pulmonary fibrosis 73, radiation-induced liver damage 74, brain damage 75, and salivary gland damage 76. More research predominantly focuses on radiation-induced intestinal injury 77-80. Similarly, investigations on IR-induced ferroptosis in radiation injury have primarily concentrated on intestinal injury 81-84, lung injury 85-88, with limited studies on hematopoietic system injury 89, 90, wound repair 91, hippocampus neurons injury 92, and skin injury 93. Research on radiation-induced necroptosis in the field of radiation injury primarily focuses on lung injury 94 and intestinal injury 95.

**Figure 1:**
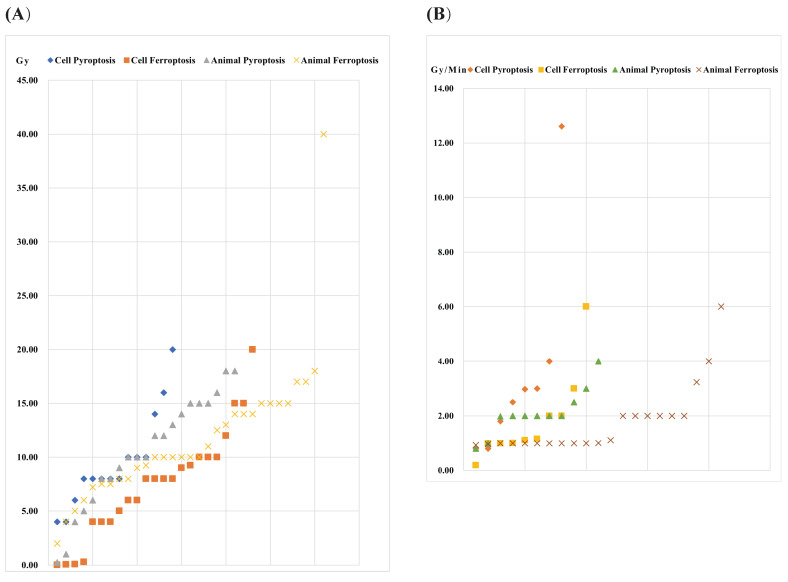
** IR dose for pyroptosis- and ferroptosis-related radiation injury.** (A) Total dose (B) Dose rate.

The occurrence of pyroptosis, ferroptosis and necroptosis induced by IR is dependent on the dose and dose rate. Generally, the total IR dose for both pyroptosis, ferroptosis and necroptosis is below 10 Gy, occasionally ranging from 10-25 Gy. Notably, Wang et al. employed a higher dose of 40 Gy to induce ferroptosis, establishing a radiation-induced skin injury model in Sprague-Dawley (SD) rats 93. Compared with ferroptosis and necroptosis, pyroptosis requires a larger total IR dose. For ferroptosis and necroptosis, the administered dose to animals is relatively higher than that given to cells, with no obvious trend observed in pyroptosis **(Figure 1) (Supplementary Table 1)**. Regarding dose rates, 1-4 Gy/min is commonly applied. Zhang et al. conducted a study using a high dose rate of 12.61 Gy/min to induce pyroptosis 96. No distinct pattern emerges between cell death modes and cell/animal models. However, parameters such as total dose, fractionation, and dose rate should be considered when constructing a radiation injury model. Additionally, animal factors, including the use of C57BL/6J mice, ICR mice, SD rats, and Balb/c mice, should be taken into account. Due to variations in radiation sensitivities, the IR parameters used to induce damage in different organs vary. The doses for radiation injury induction are often higher than those for sensitivity enhancement. In animal models, there is no clear pattern regarding the radiation dose inducing pyroptosis, ferroptosis or necroptosis, and the type of cell death produced by similar doses is uncertain. Common parameters of radiation injury caused by pyroptosis, ferroptosis and necroptosis are presented in **Supplementary Table 1**.

### Regulation of radiation induced-pyroptosis and its mechanisms

Various agents, including biological products/extracts, nuclear factor kappa-B (NF-κB) inhibitors, and different compounds, can inhibit radiation-induced pyroptosis and mitigate radiation injury. In **Supplementary Table 2**, we have compiled a summary of pyroptosis inhibitors that provide protection against radiation injury, along with elucidation of their underlying mechanisms.

**Figure 2:**
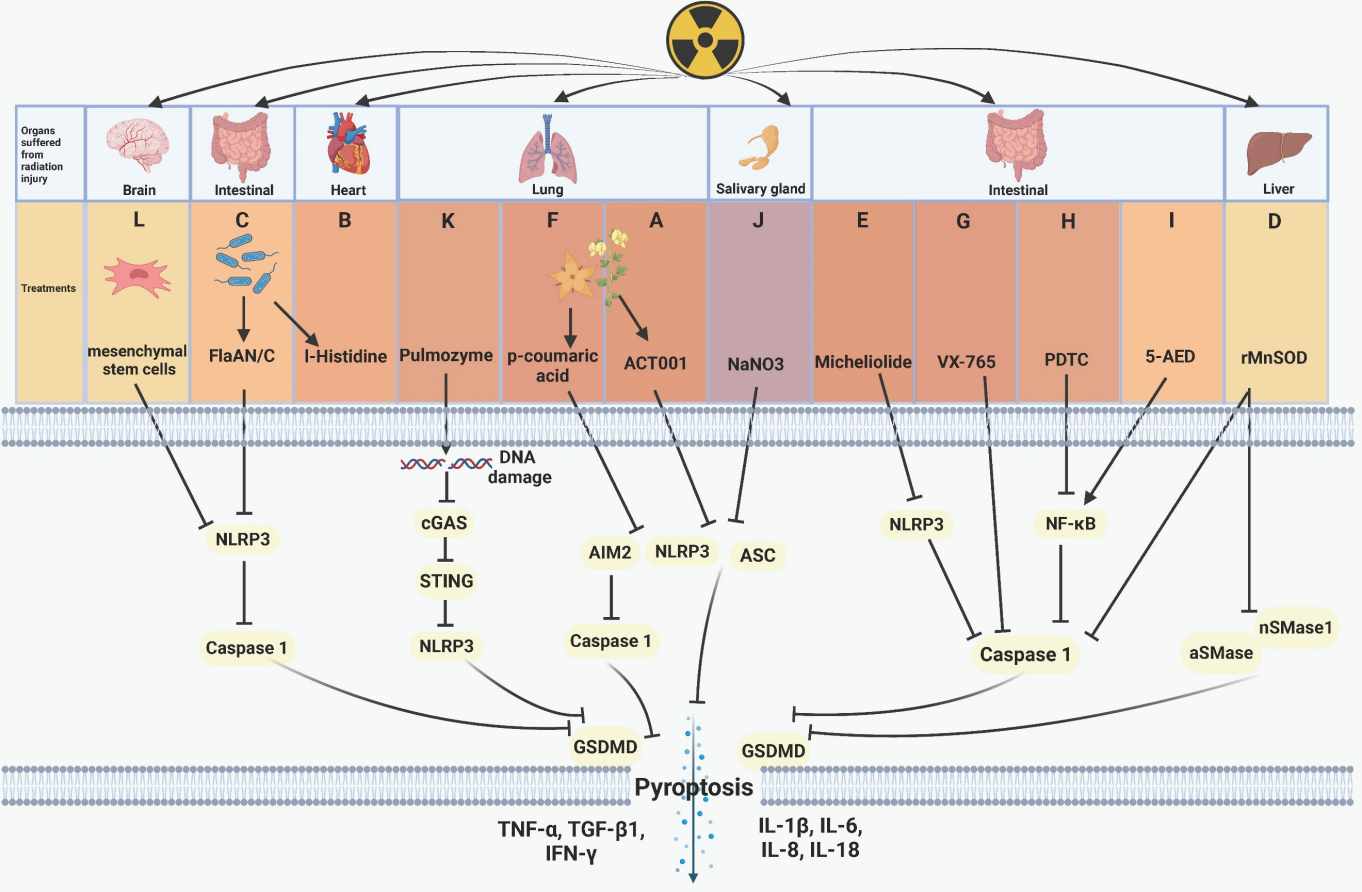
** Regulative mechanism of inhibitors for treating pyroptosis-related radiation injury.** (A) The feverfew plant extract ACT001 inhibits NLRP3 and reduces the expression of IL-6, TNF-α, IL-1β, and GSDMD, thereby inhibiting pyroptosis and radiation-induced lung injury. (B) I-Histidine, secreted by intestinal flora, mitigates radiation-induced cardiopulmonary injury by inhibiting pyroptosis. (C) Bacterial-derived FlaAN/C inhibits ROS, NLRP3, and caspase-1, thus attenuating intestinal cells pyroptosis and the release of inflammation-related cytokines, including IL-1β, IL-18, IL-8, IL-6, and TNF-α. (D) rMnSOD inhibits radiation-induced liver damage by inactivating caspase-1, aSMase, and nSMases. (E) Micheliolide inhibits the NLRP3/caspase-1 axis, attenuating pyroptosis and the release of cytokines including IL-1β, IL-18, TGF-β1, TNF-α, and IFN-γ, which ultimately promots mice survival after radiation exposure. (F) p-Coumaric acid inactivates caspase-1, NLRP3, and AIM2 to inhibit pyroptosis and radiation-induced lung injury. (G-H) VX-765 and the NF-κB inhibitor PDTC inhibit caspase-1 to alleviate radiation-induced intestinal damage. (I) 5-AED upregulates the expression of NF-κB to mitigate AIM2-driven pyroptosis and radiation-induced intestinal injury. (J) NaNO3 inhibits pyroptosis and acinar cells damage by downregulating the expression of NLPR3, ASC, GSDMD, IL-18, thus inhibiting pyroptosis. (K) Pulmozyme inhibits double-strand DNA damage and the the cGAS/STING/NLRP3 axis activation and the release of IL-1β, IL-18, protecting lung tissue from radiation damage. (L) Mesenchymal stem cells inhibit NLRP3 and caspase-1 to alleviate radiation-induced brain damage.

Plant extracts or microbial products have shown inhibitory effects on pyroptosis, functioning as radiation protection factors. For instance, ACT001, derived from parthenolide found in the feverfew plant, inhibits NOD-like receptor thermal protein domain associated protein 3 (NLRP3) and reduces the expression of IL-1β, interleukin-6 (IL-6), tumor necrosis factor-α (TNF-α), and GSDMD. This ultimately inhibits pyroptosis and radiation-induced lung injury **(Figure 2A)** 73. Andrographis paniculate extract and its active compound andrographolide, along with the traditional Chinese medicine Re-Du-Ning (RDN) demonstrate pronounced efficacy in preventing absent in melanoma 2 (AIM2) from sensing DNA damage caused by IR. These agents effectively pyroptosis-induced pneumonia and suppress epithelial-mesenchymal transition (EMT)-mediated pulmonary fibrosis 97, 98. Cui et al. discovered that I-Histidine, secreted by intestinal flora, and its metabolite imidazole propionate attenuate radiation toxicity in the heart and lungs by inhibiting pyroptosis **(Figure 2B)** 99. Additionally, Xu's team confirmed that bacterially derived flagellin A N/C (FlaAN/C) contributes to the restoration of intestinal vitiligo and the reduction of hemorrhage areas by inhibiting pyroptosis and apoptosis. FlaAN/C significantly inhibits ROS, NLRP3, and caspase-1 in intestinal cells post-IR, subsequently diminishing the release of inflammation-related cytokines such as IL-1β, IL-6, IL-18, interleukin-8 (IL-8), and TNF-α **(Figure 2C)** 80. Biological extracts have emerged as significant agents for suppressing pyroptosis related radiation injury.

Numerous studies proved that the NF-κB pathway contributes to the development of pyroptosis 100, 101. Commonly used NF-κB inhibitors include andrographolide 97, human recombinant manganese superoxide dismutase (rMnSOD) 74, micheliolide 92, p-Coumaric acid 79, and pyrrolidinedithiocarbamate ammonium (PDTC) 102. Pre-administration of these inhibitors followed by irradiation effectively reduces cell pyroptosis and the release of pro-inflammatory factors, thereby mitigating radiation injury. However, they do not directly affect NF-κB pathway but interferes with different molecules on the pyroptosis pathway. For example, andrographolide 97 and RDN 98 effectively prevent AIM2-mediated pyroptosis-related radiation pneumonitis. rMnSOD inhibits radiation-induced hepatocyte pyroptosis by reducing the activity of elevated caspase-1, acid sphingomyelinases (aSMase), and neutral sphingomyelinases (nSMases) **(Figure 2D)** 74. Micheliolide facilitates the phagocytosis of NLRP3, reduces NLRP3 expression, and prolongs the survival of mice after radiation exposure **(Figure 2E)** 77. p-Coumaric acid inhibits both pyroptosis and apoptosis while enhancing intestinal protection function. Mechanistically, p-Coumaric acid downregulates the activities of caspase-1, NLRP3, AIM2, concurrently promoting the expression of tight junction proteins, including occludin, zonula occludens-1 (ZO-1), and claudin-5 **(Figure 2F)** 79. Xu et al. demonstrated the radioprotective ability of FlaAN/C 80 and further showed that miR-142a-3p downregulates interleukin 1 receptor associated kinase 1 (IRAK1), impedes the NF-κB pathway, and amplifies the protective effect against radiation-induced intestinal injury 103. Zhang et al. demonstrated that the caspase-1 inhibitor VX-765 **(Figure 2G)** and the ferroptosis inhibitor ferrostatin-1 hindered pyroptosis and ferroptosis, respectively. PDTC could simultaneously reverse indicators of pyroptosis and ferroptosis by inhibiting NF-κB **(Figure 2H)** 102. Overall, inhibiting NF-κB can suppress radiation-induced pyroptosis and mitigate radiation injury. However, Zhu's team reported that 5-androstenediol (5-AED) increased the expression of NF-κB and alleviated AIM2-driven pyroptosis-related intestinal injury **(Figure 2I)** 78. The NF-κB pathway has been demonstrated as a target of pyroptosis to govern radiation injury.

**Figure 3:**
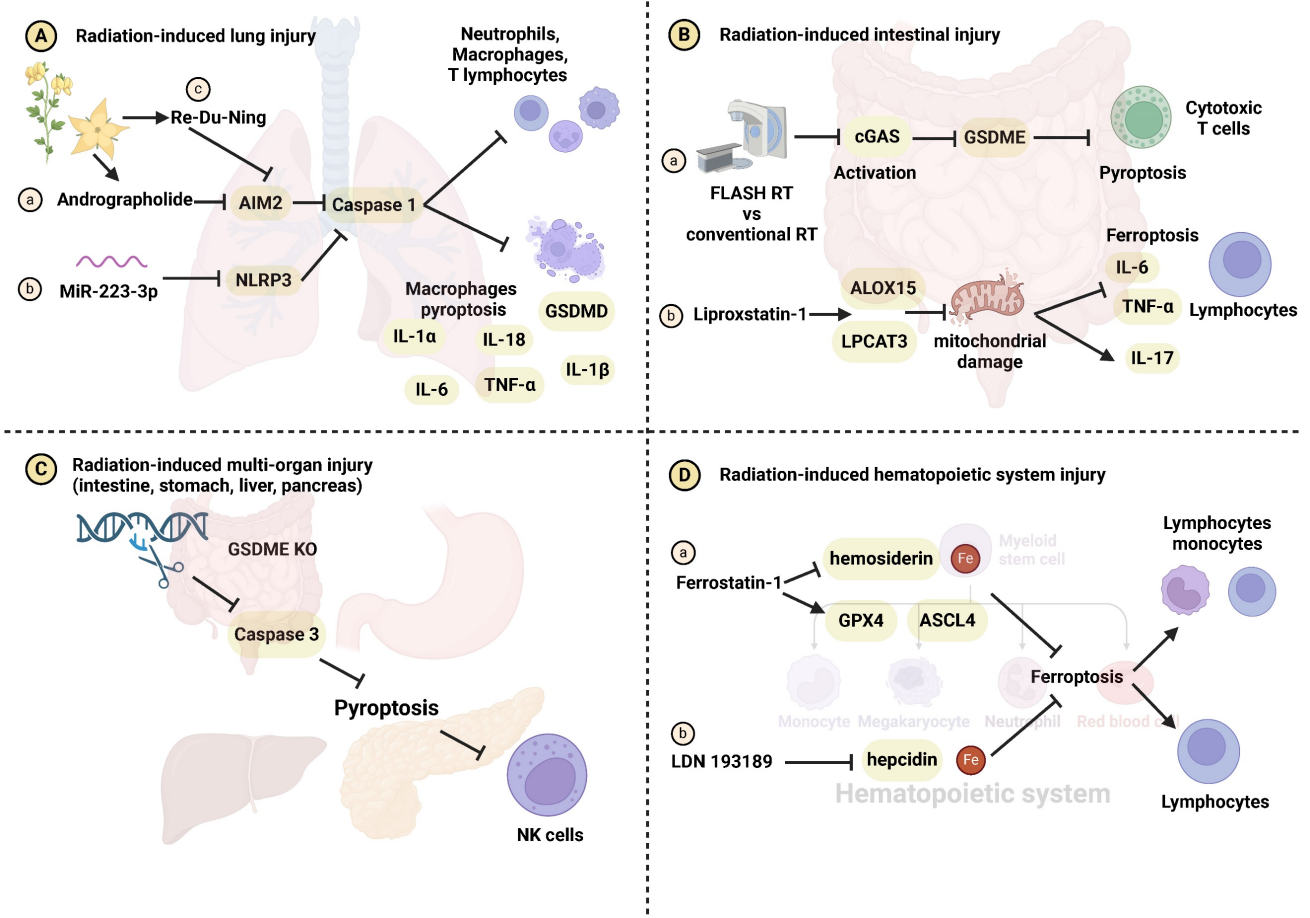
** The role of immune regulation in pyroptosis- and ferroptosis-related radiation damage.** (A) Radiation-induced lung injury. (Aa-b) Andrographolide and miR-223-3p inhibit macrophage pyroptosis, the release of IL-1α, IL-1β, IL-6, IL-18, TNF-α and radiation-induced lung injury through the AIM2/caspase-1 and NLRP3/caspase-1 axes, respectively. (Ac) RDN inhibits the AIM2/caspase-1 axis, reducing the recruitment of immune cells such as macrophages, neutrophils, and T lymphocytes, and mitigating radiation-induced lung injury. (B) Radiation-induced intestinal injury. (Ba) FLASH-RT inhibits the cGAS-STING pathway, attenuating GSDME-induced pyroptosis and cytotoxic T cells infiltration, ultimately protecting intestinal cells from radiation damage. (Bb) Liproxstatin-1 upregulates LPCAT3 and ALOX15 expression and inhibits mitochondrial damage, ultimately preventing ferroptosis, the release of IL-6, IL-17, TNF-α and lymphocytes infiltration. (C) Radiation-induced multi-organ (intestine, stomach, liver and pancreas) injury. Inhibition of pyroptosis by regulating the GSDME/caspase-3 axis blocks NK cells recruitment, mitigating radiation-induced multi-organ damage. (D) Radiation-induced hematopoietic system injury. (Da-b) Ferrostatin-1 and LDN 193189 inhibit iron metabolism to protect the hematopoietic system from radiation damage.

Alterations in the immune microenvironment pre and post RT contribute to the development of radiation-induced damage 104, 105. In contrast to apoptosis, known for immune silencing, pyroptosis disrupts the cell membrane and releases cellular contents, triggering immune activation 41, 106. The impact of IR on the immune system has garnered substantial attention 107, 108. Han et al. reported that IR induces pyroptosis and recruits cytotoxic T cells through the caspase-9/caspase-3/gasdermin E (GSDME) signaling pathway 109. Combining IR with chemotherapy drugs like cisplatin, etoposide, decitabine, and azacytidine has the potential to further enhance anti-tumor immunity 109. IR-induced pyroptosis can influence radiation-induced damage development by modulating macrophages, dendritic cells, T cells, and natural killer cells (NK cells). Andrographolide **(Figure 3Aa)** and miR-223-3p **(Figure 3Ab)** inhibit caspase-1-mediated macrophage pyroptosis by targeting the AIM2 and NLRP3 inflammasomes, thereby mitigating radiation-induced lung injury 96, 97. The combination of RDN and IR effectively prevents pyroptosis and radiation pneumonitis. Mechanistically, RDN suppresses the activation of the PI3K/AKT pathway and AIM2, blocking immune cells infiltration like macrophages, neutrophils, and T lymphocytes **(Figure 3Ac)** 98. Compared to conventional RT, FLASH-RT attenuates GSDME-mediated pyroptosis by inhibiting the cyclic GMP-AMP synthase (cGAS)-stimulator of interferon genes (STING) pathway. This reduction in activity leads to a decrease in CD8+ T cells recruitment and subsequently mitigates radiation-induced intestinal damage **(Figure 3Ba)** 110. Additionally, Wu et al. demonstrated that knocking out cGAS downregulates caspase-11-mediated pyroptosis and reduce sepsis following IR 72. Interestingly, GSDME/caspase3-induced pyroptosis and NK cells activation exhibit a dual effect, concurrently diminishing radiation resistance and increasing radiation-related organ toxicity in the intestine, liver, stomach, and pancreas **(Figure 3C)** 111. Macrophages, T cells, neutrophils, and NK cells within the immune microenvironment play crucial roles in the process of pyroptosis implicated in the development of radiation injury. The relevant molecular mechanisms have been summarized in **Supplementary Table 3**.

Other substances, such as NaNO3, pulmozyme, and mesenchymal stem cells, show potential as radioprotective agents. NaNO3 has been identified to reduce the radiation-induced expression of GSDMD, GSDMD-NT, ASC, and IL-18, thereby inhibiting NLPR3-mediated pyroptosis and protecting acinar cells from damage **(Figure 2J)** 76. Pulmozyme, a widely used clinical drug for cystic fibrosis, can inhibit double-strand break of DNA damage induced by IR and the activation of the cGAS/STING/NLRP3 signaling pathway. This inhibition subsequently prevents pyroptosis and radiation-induced lung damage **(Figure 2K)** 112. Mesenchymal stem cells possess the capability to suppress the activation of NLRP3 and caspase-1, thereby attenuating microglial pyroptosis and protecting brain tissue from radiation injury **(Figure 2L)** 75. Biological extracts, NF-κB pathway regulators, and compounds with special structures have been revealed to be effective agents targeting pyroptosis to treat radiation injury.

### Regulation of radiation induced-ferroptosis and its mechanisms

**Figure 4:**
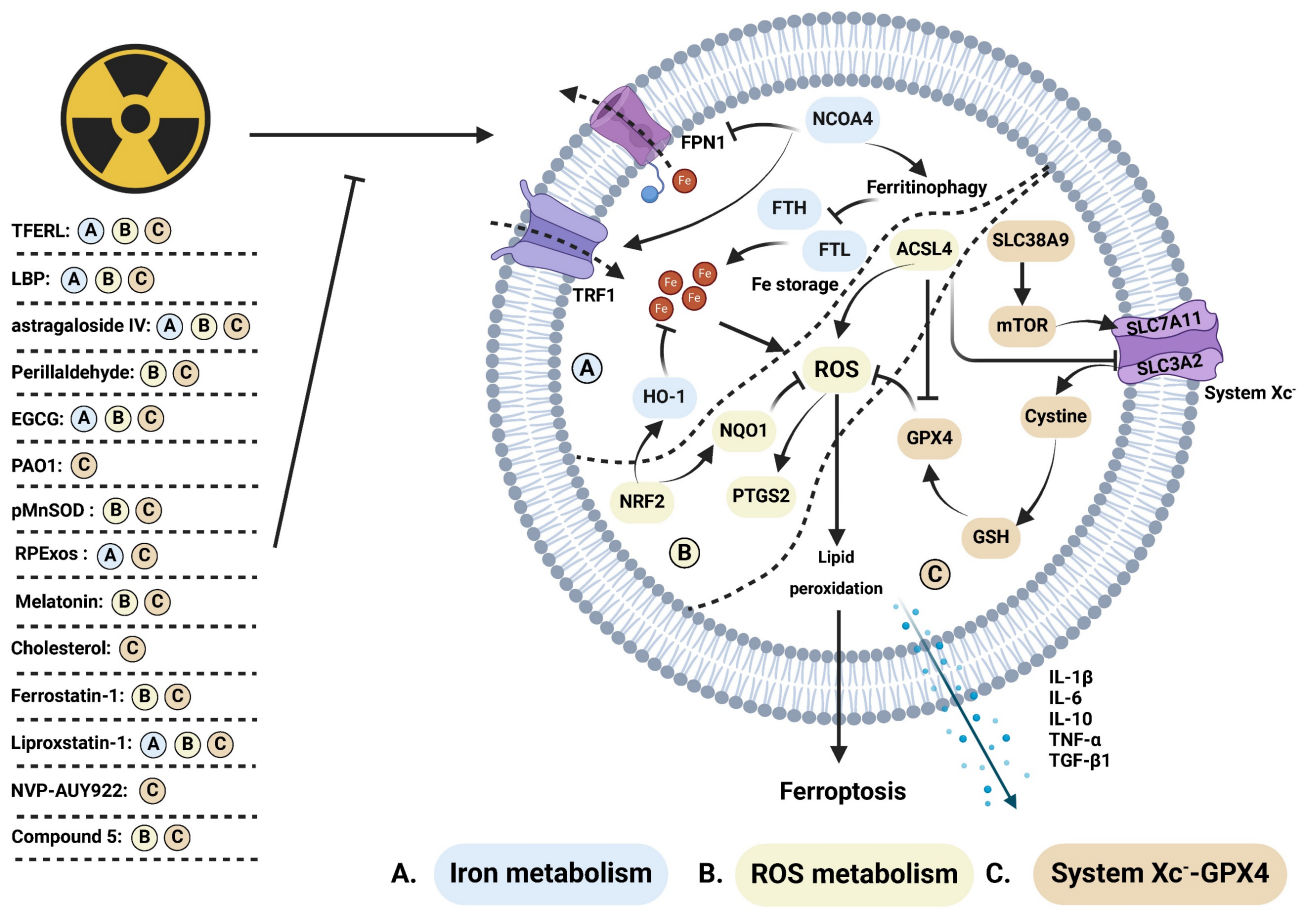
** Regulative mechanism of inhibitors for treating ferroptosis-related radiation damage.** Radiotherapy can stimulate (A) iron metabolism and (B) ROS metabolism while inhibiting (C) the system Xc^-^-GPX4 axis, ultimately inducing ferroptosis. This process is suppressed by several inhibitors. (A) Iron metabolism: NCOA4 promotes ferritinophagy, inhibiting the expression of iron storage-related proteins such as FTH and FTL, while suppressing FPN1 and enhancing TRF1, thereby synergistically promoting iron metabolism. Activation of HO-1, facilitated by NRF2, further inhibits iron metabolism. Dysregulated iron metabolism leads to ROS accumulation. (B) ROS metabolism: NRF2 upregulates NQO1, which counteracts ROS levels elevated by ACSL4 and induces increased PTGS2 expression. Elevated ROS levels promote lipid peroxidation, triggering ferroptosis. (C) System Xc^-^-GPX4: SLC38A9 activates mTOR, inhibiting system Xc^-^ comprised of SLC7A11 and SLC3A2. Cystine influx via system Xc^-^ activates the GSH/GPX4 axis, inhibiting ROS accumulation. ACSL4 indirectly inhibits system Xc^-^ and GPX4, thereby promoting ROS accumulation. Ferroptosis induction leads to the release of cytokines, including IL-1β, IL-6, IL-10, TNF-α, and TGF-β1.

Plant extracts exhibit radioprotective effects by inhibiting ferroptosis **(Figure 4)**. Astragaloside IV (AS IV), a saponin derived from astragalus, mitigates radiation-induced lung injury by inhibiting ferroptosis 85. Lycium barbarum polysaccharide-glycoprotein (LBP), obtained from Lycium barbarum fruit, protects epithelial cells from radiation injury by upregulating nuclear factor erythroid-2 related factor 2 (NRF2) and solute carrier family 7 member 11 (SLC7A11), which are positive regulators of cell survival via ferroptosis 113. Epigallocatechin-3-gallate (EGCG), a key extract from green tea, promotes the survival of intestinal stem cells and mucosal cells by inhibiting both ferroptosis and apoptosis. Mechanistically, EGCG enhances NRF2 expression and facilitates its nuclear translocation, resulting in the upregulation of SLC7A11, HO-1, and glutathione peroxidase 4 (GPX4), while reducing ROS accumulation and DNA damage 114. Similarly, perillaldehyde, the active ingredient of perilla frutescence, upregulates NRF2, GPX4, and glutathione (GSH), while inhibiting ROS accumulation and prostaglandin-endoperoxide synthase 2 (PTGS2) to prevent ferroptosis, apoptosis, and radiation-induced intestinal barrier damage 115. Notably, total flavonoids derived from Engelhardia roxburghiana Wall. leaves (TFERL) alleviate radiation-induced intestinal damage in mice and reduce the radiation resistance of colorectal cancer cells by inhibiting ferroptosis 116. Ferroptosis inhibitors safeguarding against radiation injury, along with their corresponding mechanisms, are summarized in **Supplementary Table 2**.

Exosomes and plasmids serve as important carriers for cell-free therapies, gaining notable attention in recent years 117, 118. Ferroptosis plays a crucial role in repairing radiation-induced damage to the hematopoietic system and skin. Yao et al. reported that the injecting exosomes derived from healthy rats' plasma (RPExos) promoted fibroblast growth and wound healing in mouse skin injuries caused by irradiation 91. Additionally, plasmid-loaded human MnSOD downregulates acyl-CoA synthetase long-chain family member 4 (ACSL4) and reduces ROS production. It concurrently upregulates the expression of GPX4 and SLC7A11 to attenuate ferroptosis and radiation-induced skin damage 93. Exosomes and plasmids hold promise as important delivery vehicles for targeting ferroptosis in the management of radiation injury.

Melatonin and cholesterol have demonstrated radioprotective potential in preclinical studies **(Figure 4)**. Du's team reported that melatonin, secreted by the pineal gland, promotes the binding of NRF2 to pyruvate kinase isozymes M2 (PKM2) and facilitates its nuclear translocation. This protective mechanism mitigates radiation-induced hippocampal neuron death by attenuating ferroptosis 92. Cholesterol enhances the ferroptosis resistance of bone marrow hematopoietic stem cells **(Figure 4)**. This is achieved through the activation of the solute carrier family 38 member 9 (SLC38A9)/mammalian target of rapamycin (mTOR) axis and SLC7A11/GPX4 axis, along with the inhibition of ferritinophagy. These mechanisms collectively attenuate radiation-induced myelosuppression 119. Conversely, Tyurina et al. reported that pseudomonas aeruginosa (PAO1) induces ferroptosis by promoting the generation of 15-HpETE-PE and oxidized phosphatidylethanolamine through 15-lipoxygenase. This further enhances intestinal damage 83. Human endogenous metabolites could serve as crucial components of radioprotectants by exerting inhibitory effects on ferroptosis.

Ferroptosis inhibitors, such as ferrostatin-1, deferoxamine (DFO), liproxstatin-1, NVP-AUY922, and compound 5, have demonstrated significant efficacy in inhibiting ferroptosis and alleviating radiation injury **(Figure 4)**. IR stimulates the STAT1/interferon regulatory factor (IRF1)/ACSL4 signaling pathway and nuclear receptor coactivator 4 (NCOA4)-induced ferritinophagy, leading to intracellular iron overload and ferroptosis. Ferrostatin-1 and the DFO effectively attenuate iron metabolism, thereby alleviating radiation-induced intestinal injury 81, 82. Additionally, Li et al. reported that ferrostatin-1 enhances radiation sensitivity while reducing radiation-induced intestinal injury 120. Liproxstatin-1, as a ferroptosis inhibitor, increases the expression of NRF2, heme oxygenase-1 (HO1), and NADH quinone oxidoreductase 1 (NQO1), while concurrently inhibiting the expression of transforming growth factor-β (TGF-β1). Qiao et al. demonstrated that liproxstatin-1 reduces the release of TNF-α, IL-6, and IL-10, ultimately alleviating radiation-induced lung injury 87, 88. Furthermore, NVP-AUY922, a resorcinol isoxazole amide drug with anti-inflammatory, immunomodulatory, and therapeutic effects on various cancers. It inhibits the heat shock protein (HSP90)-mediated CMA pathway, promotes chaperone-mediated lysosomal degradation of GPX4, ultimately inhibits ferroptosis, inflammatory factor release, and radiation-induced lung injury. Tian et al. synthesized a series of modified polycysteine peptides to demonstrate the binding ability of drugs to DNA. Compound 5, in particular, reduces hematopoietic system damage and lung damage caused by whole-body irradiation. It also inhibits NADPH oxidase 1 (NOX1), ferroptosis, and alleviates radiation-induced intestinal damage 121. ACSL4 levels elevate post-IR, subsequently inducing ferroptosis and intestinal injury. Dai's team utilized antibiotics and antifungal drugs to inhibit ACSL4 expression and mitigate intestinal injury, proving the involvement of intestinal flora and ACSL4 in inducing ferroptosis and intestinal injury 84. Several ferroptosis inhibitors frequently utilized in preclinical investigations hold promise as emerging radioprotective agents.

Emerging evidence indicates a close relationship between radiation-induced ferroptosis and immune system activation. Hu et al. demonstrated that the restoration of radiation-induced intestinal immune imbalance by the ferroptosis inhibitor liproxstatin-1 **(Figure 3Bb)** 122. Furthermore, ferrostatin-1 **(Figure 3Da)**, LDN 193189 **(Figure 3Db)**, and DFO have been shown to reduce bone marrow suppression and restore the number of red/white blood cells by inhibiting iron metabolism. Consequently, this intervention alleviates acute radiation symptoms such as bleeding and prolongs mouse survival 89, 90. Additionally, Ding et al. observed ferroptosis induction in AHH-1 lymphocytes following exposure to low-dose radiation (0-4.7 mGy) 123. However, the expression of ferroptosis markers decreased after 4.8-28.8 mGy irradiation. Lymphocytes and monocytes, integral components of the immune microenvironment, have been identified as crucial mediators in the development of radiation injury triggered by ferroptosis.

### Regulation of radiation induced-necroptosis and its mechanisms

**Figure 5:**
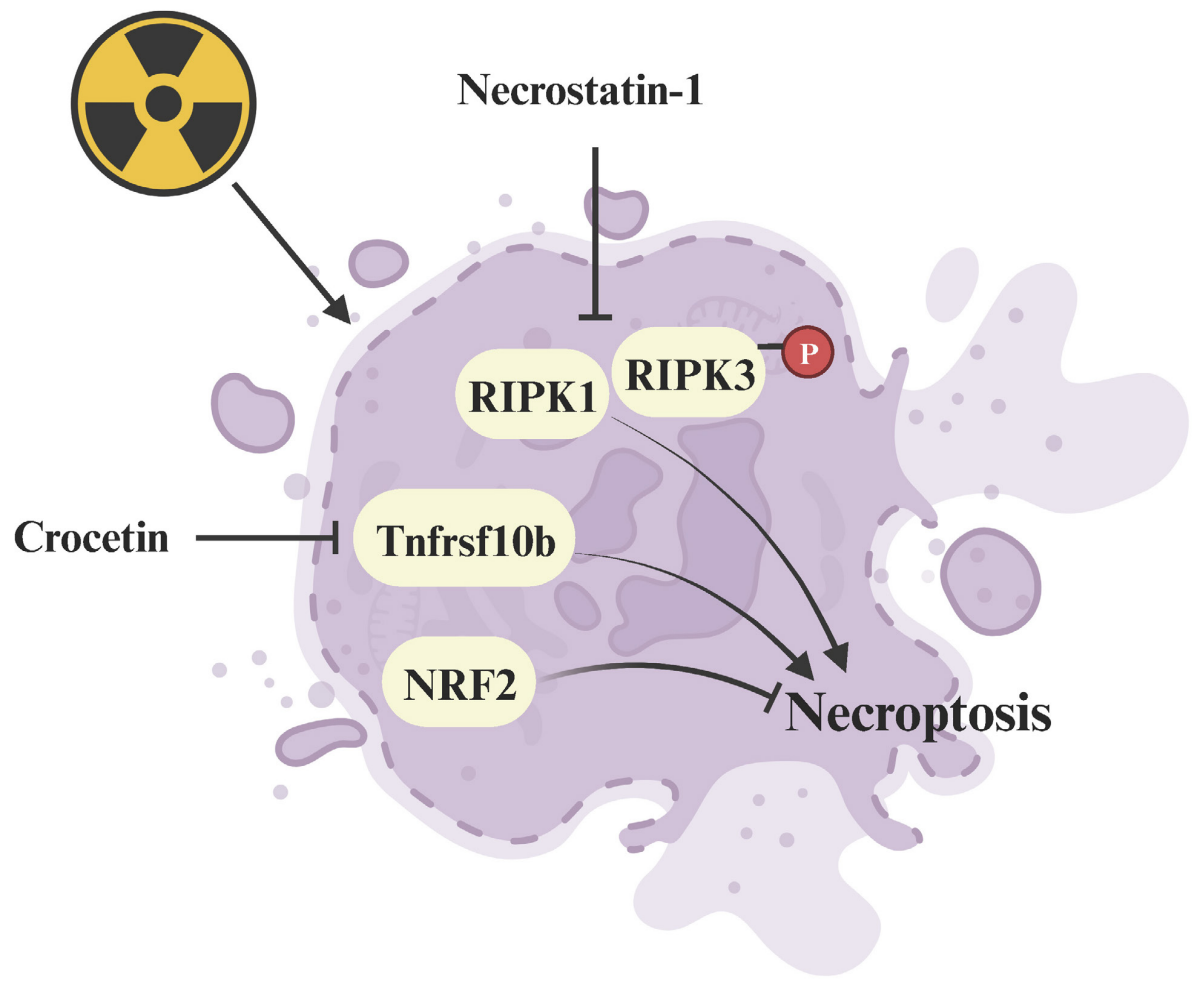
** Regulative mechanism of inhibitors for treating necroptosis-related radiation damage.** Crocetin and necrostatin-1 inhibit necroptosis by suppressing the radiation-induced upregulation of Tnfrsf10b and RIPK1/p-RIPK3 expression respectively.

Plant extracts, such as crocetin derived from gardenia fruit and lipoxygenase-15 derived from baicalein, exhibit potential as radioprotective drugs **(Figure 5)**. Crocetin alleviates radiation injury to lung tissue structure by inhibiting the expression of Tnfrsf10b, thereby hindering the occurrence of necroptosis 94. Greenberger et al. reported that lipoxygenase-15, along with the classic necroptosis inhibitor necrostatin-1 can inhibit necroptosis and extend the survival of radiation-damaged animals 124. Additionally, Kagan's team demonstrated that necrostatin-1 **(Figure 5)** inhibits necroptosis and counters radiation-induced lethal damage by suppressing the expression of receptor-interacting serine/threonine-protein kinase 1 (RIPK1) and phosphorylating RIPK3 125. Furthermore, NRF2, a key regulator of ferroptosis, has been identified as a suppressor of necroptosis, alleviating radiation-induced rectal injury 95. Agents with the capacity to suppress necroptosis show promise as innovative radioprotective therapies. Necroptosis inhibitors, along with their protective mechanisms against radiation injury are summarized in **Supplementary Table 2**.

### Clinical translations

In recent years, clinical trials studying pyroptosis, ferroptosis and necroptosis have emerged. However, trials specifically targeting pyroptosis, ferroptosis, necroptosis, and radiation injury are rare. Wang et al. summarized clinical trials focusing on pyroptosis, ferroptosis, and necroptosis in the context of RT 126. Most of these clinical trials are in phase I or II, with few in phase III. Nevertheless, their emphasis lies predominantly on alterations in the expression of key regulatory molecules associated with pyroptosis, ferroptosis, and necroptosis, which may not comprehensively depict the incidence of cell death 126. Presently, clinical trials addressing radiation injury primarily concentrate on radiation dermatitis 127-129, with a limited number focusing on radiation pneumonitis 130 and radiation enteritis 131. The majority of interventions remain symptomatic, with approaches like hyperbaric oxygen therapy, administration of glucocorticoid, pain management, and nutritional support 13, 14, 132-134. The clinical efficacy of therapies targeting cell death for radiation injury treatment is yet to be substantiated. Currently, only a sparse number of clinical trials are investigating drugs with potential cell death inhibitory effects in the context of radiation injury, such as enalapril and captopril 135-138. The actual impact of these drugs on improving radiation injury through the inhibition of cell death necessitates further confirmation.

## Conclusion and prospective

Radiotherapy (RT) is a double-edged sword. While it effectively kills tumor cells, it also damages surrounding normal tissues, causing radiation injury 139, 140. Cell death serves as one of the mechanisms that explains both phenomena. This review focuses on the roles of pyroptosis, ferroptosis, and necroptosis in the radiation injury process, attracting significant attention among oncologists.

Overall, the occurrence of pyroptosis, ferroptosis, and necroptosis plays a significant role in mediating radiation injury to a certain extent. Most studies on pyroptosis, ferroptosis, and necroptosis in the field of radiation injury use combined inducers or inhibitors, such as biological extracts and NF-κB inhibitors. Current investigations into radiation injury to normal tissue do not concurrently address the radiation resistance of tumor tissue, deviating from the actual clinical scenario. However, a few studies have indicated that ferroptosis is inhibited under radiation, potentially associated with tumor type and radiation dose. Similar to autophagy, ferroptosis may protect tumors. Ma et al. reported that IR activates the AdipoR1/NRF2 signaling pathway to inhibit ferroptosis, thereby reducing the radioresistance of liver cancer cells 141. However, no relevant evidence exists in the field of radiation injury, and further research is needed to confirm this. Research on IR-induced necroptosis in the context of radiation injury is scarce, with poorly understood underlying mechanisms.

In most studies, identifying pyroptosis, ferroptosis, and necroptosis primarily relies on changes in classic targets, with limited research on the specific upstream and downstream mechanisms. Evidence suggests potential intrinsic connections between different cell death pathways 142, 143. While radiation can induce multiple forms of cell death simultaneously 144, 145, research on the transformation between these death modes is lacking. The upregulation of the NFR2 induced by radiation is implicated in the occurrence and progression of ferroptosis and necroptosis 95, 102, 146. Research on cell death is continuously evolving. Whether other forms of cell death, such as cuproptosis, alkaliptosis, parthanatos, oxeiptosis, and disulfidptosis, are involved in the occurrence of radiation damage requires additional evidence.

Currently, there are limited clinically effective radiation protection drugs. Inhibitors related to pyroptosis, ferroptosis, and necroptosis have been summarized, and preclinical studies have shown their potential in attenuating radiation injury. However, further clinical trials are necessary for more credible evidence. Wang et al. summarized the biomarkers of pyroptosis, ferroptosis, and necroptosis, but most of these studies rely on single characteristic molecules to identify specific forms of cell death, which may not fully reflect their actual impact in RT 126. Detecting specific death modes during treatment remains a concern. Drugs with cell death inhibitory effects, such as tetrahydrobiopterin 147, enalapril 138, captopril 135, fenofibrate 148, etc., are being investigated in clinical trials related to radiation injury. However, it is yet to be proven whether the efficacy of these drugs, developed to treat radiation injury, is primarily attributed to inhibiting cell death. The clinical translation of therapies based on cell death modulation is still in the developmental stage.

In clinical settings, radiation injury occurs when normal tissues receive radiation doses intended for non-tumor target areas. However, preclinical models often employ high radiation doses to induce damage, not accurately reflecting the clinical situation. Most studies only mention the total exposure dose in the dosimetry section, lacking information on exposure dose rate, fractionation, and exposure time intervals. To facilitate further progress in research, we provide detailed evidence on the establishment of animal models and radiation injury mechanisms. Some studies may have conflicting dosimetry 109, 149, likely attributed to heterogeneity across studies. Furthermore, due to species differences, there is still a considerable gap before research findings from preclinical models can be translated into clinical practice. FLASH, proton, and heavy ion RT are innovative RT methods aiming to spare normal tissues while effectively targeting tumor cells. Studies have shown that compared to conventional therapy, FLASH-RT induces lower levels of pyroptosis and intestinal injury 110. Investigating pyroptosis, ferroptosis and necroptosis can unveil the protective mechanisms of FLASH, proton, and heavy ion RT on normal tissues.

This article provides a comprehensive review of the involvement of pyroptosis, ferroptosis, and necroptosis in radiation injury generation and their underlying mechanisms. A particular emphasis is placed on the relevant dosimetry, serving as an important reference for future studies. The use of cell death inhibitors specific to these pathways holds promise for providing radioprotective effects. Further research in this field will contribute to establishing a robust theoretical foundation for effectively harnessing pyroptosis, ferroptosis, and necroptosis to mitigate radiation injury.

## Supplementary Material

Supplementary tables.

## Abbreviations

RT: radiotherapy
IR: ionizing radiation
AIM2: prevent absent in melanoma 2
NF-κB: nuclear factor kappa-B
GSDMD: gasdermin D
NLRP3: NOD-like receptor thermal protein domain associated protein 3
ROS: reactive oxygen species
NRF2: nuclear factor erythroid-2 related factor 2
SLC7A11: solute carrier family 7 member 11
GPX4: glutathione peroxidase 4
ACSL4: acyl-CoA synthetase long-chain family member 4

## References

[1] Smith BD, Haffty BG, Wilson LD, Smith GL, Patel AN, Buchholz TA (2010). The future of radiation oncology in the United States from 2010 to 2020: will supply keep pace with demand?. Journal of clinical oncology: official journal of the American Society of Clinical Oncology.

[2] Huang Y, Wang H, Luo G, Zhang Y, Wang L, Li K (2017). A systematic review and network meta-analysis of neoadjuvant therapy combined with surgery for patients with resectable esophageal squamous cell carcinoma. International journal of surgery (London, England).

[3] Gillen S, Schuster T, Meyer Zum Büschenfelde C, Friess H, Kleeff J (2010). Preoperative/neoadjuvant therapy in pancreatic cancer: a systematic review and meta-analysis of response and resection percentages. PLoS medicine.

[4] Tallet AV, Dhermain F, Le Rhun E, Noël G, Kirova YM (2017). Combined irradiation and targeted therapy or immune checkpoint blockade in brain metastases: toxicities and efficacy. Annals of oncology: official journal of the European Society for Medical Oncology.

[5] Pitroda SP, Chmura SJ, Weichselbaum RR (2019). Integration of radiotherapy and immunotherapy for treatment of oligometastases. The Lancet Oncology.

[6] Thoms J, Bristow RG (2010). DNA repair targeting and radiotherapy: a focus on the therapeutic ratio. Seminars in radiation oncology.

[7] Augenstein LG (1963). RADIOBIOLOGICAL MECHANISMS: COMPARATIVE DISTRIBUTION AND ROLE OF IONIZATION, EXCITATION, AND ENERGY AND CHARGE MIGRATION. Progress in biophysics and molecular biology.

[8] Lhuillier C, Rudqvist NP, Yamazaki T, Zhang T, Charpentier M, Galluzzi L (2021). Radiotherapy-exposed CD8+ and CD4+ neoantigens enhance tumor control. The Journal of clinical investigation.

[9] Hanania AN, Mainwaring W, Ghebre YT, Hanania NA, Ludwig M (2019). Radiation-Induced Lung Injury: Assessment and Management. Chest.

[10] Wang H, Wei J, Zheng Q, Meng L, Xin Y, Yin X (2019). Radiation-induced heart disease: a review of classification, mechanism and prevention. International journal of biological sciences.

[11] Balentova S, Adamkov M (2015). Molecular, Cellular and Functional Effects of Radiation-Induced Brain Injury: A Review. International journal of molecular sciences.

[12] Nguyen NP, Antoine JE, Dutta S, Karlsson U, Sallah S (2002). Current concepts in radiation enteritis and implications for future clinical trials. Cancer.

[13] Wang K, Tepper JE (2021). Radiation therapy-associated toxicity: Etiology, management, and prevention. CA: a cancer journal for clinicians.

[14] Liu L, Liang Z, Ma S, Li L, Liu X (2023). Radioprotective countermeasures for radiation injury (Review). Molecular medicine reports.

[15] Scampoli C, Cammelli S, Galietta E, Siepe G, Buwenge M, Macchia G (2022). Memantine in the Prevention of Radiation-Induced Brain Damage: A Narrative Review. Cancers.

[16] Narra VR, Howell RW, Sastry KS, Rao DV (1993). Vitamin C as a radioprotector against iodine-131 in vivo. Journal of nuclear medicine: official publication, Society of Nuclear Medicine.

[17] Ormsby RJ, Lawrence MD, Blyth BJ, Bexis K, Bezak E, Murley JS (2014). Protection from radiation-induced apoptosis by the radioprotector amifostine (WR-2721) is radiation dose dependent. Cell biology and toxicology.

[18] NCT00040365.

[19] Vijayalaxmi Reiter RJ, Tan DX Herman TS, Thomas CR Jr (2004). Melatonin as a radioprotective agent: a review. International journal of radiation oncology, biology, physics.

[20] NCT03716583.

[21] Mun GI, Kim S, Choi E, Kim CS, Lee YS (2018). Pharmacology of natural radioprotectors. Archives of pharmacal research.

[22] NCT02334345.

[23] Willemink MJ, Persson M, Pourmorteza A, Pelc NJ, Fleischmann D (2018). Photon-counting CT: Technical Principles and Clinical Prospects. Radiology.

[24] Hughes JR, Parsons JL (2020). FLASH Radiotherapy: Current Knowledge and Future Insights Using Proton-Beam Therapy. International journal of molecular sciences.

[25] Montay-Gruel P, Petersson K, Jaccard M, Boivin G, Germond JF, Petit B (2017). Irradiation in a flash: Unique sparing of memory in mice after whole brain irradiation with dose rates above 100Gy/s. Radiotherapy and oncology: journal of the European Society for Therapeutic Radiology and Oncology.

[26] Montay-Gruel P, Bouchet A, Jaccard M, Patin D, Serduc R, Aim W (2018). X-rays can trigger the FLASH effect: Ultra-high dose-rate synchrotron light source prevents normal brain injury after whole brain irradiation in mice. Radiotherapy and oncology: journal of the European Society for Therapeutic Radiology and Oncology.

[27] Alaghband Y, Cheeks SN, Allen BD, Montay-Gruel P, Doan NL, Petit B (2020). Neuroprotection of Radiosensitive Juvenile Mice by Ultra-High Dose Rate FLASH Irradiation. Cancers.

[28] Favaudon V, Caplier L, Monceau V, Pouzoulet F, Sayarath M, Fouillade C (2014). Ultrahigh dose-rate FLASH irradiation increases the differential response between normal and tumor tissue in mice. Science translational medicine.

[29] Bourhis J, Sozzi WJ, Jorge PG, Gaide O, Bailat C, Duclos F (2019). Treatment of a first patient with FLASH-radiotherapy. Radiotherapy and oncology: journal of the European Society for Therapeutic Radiology and Oncology.

[30] Yan S, Ngoma TA, Ngwa W, Bortfeld TR (2023). Global democratisation of proton radiotherapy. The Lancet Oncology.

[31] Liang S, Zhou G, Hu W (2022). Research Progress of Heavy Ion Radiotherapy for Non-Small-Cell Lung Cancer. International journal of molecular sciences.

[32] Khodamoradi E, Hoseini-Ghahfarokhi M, Amini P, Motevaseli E, Shabeeb D, Musa AE (2020). Targets for protection and mitigation of radiation injury. Cellular and molecular life sciences: CMLS.

[33] Yan G, Elbadawi M, Efferth T (2020). Multiple cell death modalities and their key features (Review). World Acad Sci J.

[34] Peng F, Liao M, Qin R, Zhu S, Peng C, Fu L (2022). Regulated cell death (RCD) in cancer: key pathways and targeted therapies. Signal transduction and targeted therapy.

[35] Sridharan H, Upton JW (2014). Programmed necrosis in microbial pathogenesis. Trends in microbiology.

[36] Zhu H, Sun A (2018). Programmed necrosis in heart disease: Molecular mechanisms and clinical implications. Journal of molecular and cellular cardiology.

[37] Tan Y, Chen Q, Li X, Zeng Z, Xiong W, Li G (2021). Pyroptosis: a new paradigm of cell death for fighting against cancer. Journal of experimental & clinical cancer research: CR.

[38] Shi J, Gao W, Shao F (2017). Pyroptosis: Gasdermin-Mediated Programmed Necrotic Cell Death. Trends in biochemical sciences.

[39] Mou Y, Wang J, Wu J, He D, Zhang C, Duan C (2019). Ferroptosis, a new form of cell death: opportunities and challenges in cancer. Journal of hematology & oncology.

[40] Dvoriantchikova G, Lypka KR, Adis EV, Ivanov D (2022). Multiple types of programmed necrosis such as necroptosis, pyroptosis, oxytosis/ferroptosis, and parthanatos contribute simultaneously to retinal damage after ischemia-reperfusion. Scientific reports.

[41] Bertheloot D, Latz E, Franklin BS (2021). Necroptosis, pyroptosis and apoptosis: an intricate game of cell death. Cellular & molecular immunology.

[42] Yu YQ, Gamez-Belmonte R, Patankar JV, Liebing E, Becker C (2022). The Role of Programmed Necrosis in Colorectal Cancer. Cancers.

[43] Cookson BT, Brennan MA (2001). Pro-inflammatory programmed cell death. Trends in microbiology.

[44] Man SM, Karki R, Kanneganti TD (2017). Molecular mechanisms and functions of pyroptosis, inflammatory caspases and inflammasomes in infectious diseases. Immunological reviews.

[45] Moujalled D, Strasser A, Liddell JR (2021). Molecular mechanisms of cell death in neurological diseases. Cell death and differentiation.

[46] Yarovinsky TO, Su M, Chen C, Xiang Y, Tang WH, Hwa J (2023). Pyroptosis in cardiovascular diseases: Pumping gasdermin on the fire. Seminars in immunology.

[47] Li L, Gao P, Tang X, Liu Z, Cao M, Luo R (2022). CB1R-stabilized NLRP3 inflammasome drives antipsychotics cardiotoxicity. Signal transduction and targeted therapy.

[48] Wei X, Xie F, Zhou X, Wu Y, Yan H, Liu T (2022). Role of pyroptosis in inflammation and cancer. Cellular & molecular immunology.

[49] Dixon SJ, Lemberg KM, Lamprecht MR, Skouta R, Zaitsev EM, Gleason CE (2012). Ferroptosis: an iron-dependent form of nonapoptotic cell death. Cell.

[50] Reichert CO, de Freitas FA, Sampaio-Silva J, Rokita-Rosa L, Barros PL, Levy D (2020). Ferroptosis Mechanisms Involved in Neurodegenerative Diseases. International journal of molecular sciences.

[51] Cheng H, Feng D, Li X, Gao L, Tang S, Liu W (2021). Iron deposition-induced ferroptosis in alveolar type II cells promotes the development of pulmonary fibrosis. Biochimica et biophysica acta Molecular basis of disease.

[52] Hu Z, Zhang H, Yang SK, Wu X, He D, Cao K (2019). Emerging Role of Ferroptosis in Acute Kidney Injury. Oxidative medicine and cellular longevity.

[53] Wu X, Li Y, Zhang S, Zhou X (2021). Ferroptosis as a novel therapeutic target for cardiovascular disease. Theranostics.

[54] Chen X, Kang R, Kroemer G, Tang D (2021). Broadening horizons: the role of ferroptosis in cancer. Nature reviews Clinical oncology.

[55] Degterev A, Huang Z, Boyce M, Li Y, Jagtap P, Mizushima N (2005). Chemical inhibitor of nonapoptotic cell death with therapeutic potential for ischemic brain injury. Nature chemical biology.

[56] Zhang X, Wu J, Liu Q, Li X, Li S, Chen J (2020). mtDNA-STING pathway promotes necroptosis-dependent enterocyte injury in intestinal ischemia reperfusion. Cell death & disease.

[57] Guo R, Jia X, Ding Z, Wang G, Jiang M, Li B (2022). Loss of MLKL ameliorates liver fibrosis by inhibiting hepatocyte necroptosis and hepatic stellate cell activation. Theranostics.

[58] Gao P, Cao M, Jiang X, Wang X, Zhang G, Tang X (2023). Cannabinoid Receptor 2-Centric Molecular Feedback Loop Drives Necroptosis in Diabetic Heart Injuries. Circulation.

[59] Balusu S, Horré K, Thrupp N, Craessaerts K, Snellinx A, Serneels L (2023). MEG3 activates necroptosis in human neuron xenografts modeling Alzheimer's disease. Science (New York, NY).

[60] Gong Y, Fan Z, Luo G, Yang C, Huang Q, Fan K (2019). The role of necroptosis in cancer biology and therapy. Molecular cancer.

[61] Kesavardhana S, Malireddi RKS, Kanneganti TD (2020). Caspases in Cell Death, Inflammation, and Pyroptosis. Annual review of immunology.

[62] Hsu SK, Li CY, Lin IL, Syue WJ, Chen YF, Cheng KC (2021). Inflammation-related pyroptosis, a novel programmed cell death pathway, and its crosstalk with immune therapy in cancer treatment. Theranostics.

[63] Rao Z, Zhu Y, Yang P, Chen Z, Xia Y, Qiao C (2022). Pyroptosis in inflammatory diseases and cancer. Theranostics.

[64] Yang WS, Stockwell BR (2016). Ferroptosis: Death by Lipid Peroxidation. Trends in cell biology.

[65] Li P, Jiang M, Li K, Li H, Zhou Y, Xiao X (2021). Glutathione peroxidase 4-regulated neutrophil ferroptosis induces systemic autoimmunity. Nature immunology.

[66] Koppula P, Zhuang L, Gan B (2021). Cystine transporter SLC7A11/xCT in cancer: ferroptosis, nutrient dependency, and cancer therapy. Protein & cell.

[67] Jiang L, Kon N, Li T, Wang SJ, Su T, Hibshoosh H (2015). Ferroptosis as a p53-mediated activity during tumour suppression. Nature.

[68] Niu B, Lei X, Xu Q, Ju Y, Xu D, Mao L (2022). Protecting mitochondria via inhibiting VDAC1 oligomerization alleviates ferroptosis in acetaminophen-induced acute liver injury. Cell biology and toxicology.

[69] Yan J, Wan P, Choksi S, Liu ZG (2022). Necroptosis and tumor progression. Trends in cancer.

[70] Belka C, Jendrossek V, Pruschy M, Vink S, Verheij M, Budach W (2004). Apoptosis-modulating agents in combination with radiotherapy-current status and outlook. International journal of radiation oncology, biology, physics.

[71] Wang F, Xia X, Yang C, Shen J, Mai J, Kim HC (2018). SMAD4 Gene Mutation Renders Pancreatic Cancer Resistance to Radiotherapy through Promotion of Autophagy. Clinical cancer research: an official journal of the American Association for Cancer Research.

[72] Wu M, Shi J, He S, Wang D, Zhang N, Wang Z (2021). cGAS promotes sepsis in radiotherapy of cancer by up-regulating caspase-11 signaling. Biochemical and biophysical research communications.

[73] Luo H, Liu X, Liu H, Wang Y, Xu K, Li J (2023). ACT001 Ameliorates ionizing radiation-induced lung injury by inhibiting NLRP3 inflammasome pathway. Biomedicine & pharmacotherapy = Biomedecine & pharmacotherapie.

[74] Cataldi S, Borrelli A, Ceccarini MR, Nakashidze I, Codini M, Belov O (2020). Acid and Neutral Sphingomyelinase Behavior in Radiation-Induced Liver Pyroptosis and in the Protective/Preventive Role of rMnSOD. International journal of molecular sciences.

[75] Liao H, Wang H, Rong X, Li E, Xu RH, Peng Y (2017). Mesenchymal Stem Cells Attenuate Radiation-Induced Brain Injury by Inhibiting Microglia Pyroptosis. BioMed research international.

[76] Li S, An W, Wang B, Li J, Qu Y, Zhang H (2021). Inorganic nitrate alleviates irradiation-induced salivary gland damage by inhibiting pyroptosis. Free radical biology & medicine.

[77] Wu DM, Li J, Shen R, Li J, Yu Y, Li L (2021). Autophagy Induced by Micheliolide Alleviates Acute Irradiation-Induced Intestinal Injury via Inhibition of the NLRP3 Inflammasome. Frontiers in pharmacology.

[78] Wu T, Liu W, Fan T, Zhong H, Zhou H, Guo W (2020). 5-Androstenediol prevents radiation injury in mice by promoting NF-κB signaling and inhibiting AIM2 inflammasome activation. Biomedicine & pharmacotherapy = Biomedecine & pharmacotherapie.

[79] Li YH, He Q, Chen YZ, Du YF, Guo YX, Xu JY (2021). p-Coumaric acid ameliorates ionizing radiation-induced intestinal injury through modulation of oxidative stress and pyroptosis. Life sciences.

[80] Wu D, Han R, Deng S, Liu T, Zhang T, Xie H (2018). Protective Effects of Flagellin A N/C Against Radiation-Induced NLR Pyrin Domain Containing 3 Inflammasome-Dependent Pyroptosis in Intestinal Cells. International journal of radiation oncology, biology, physics.

[81] Kong P, Yang M, Wang Y, Yu KN, Wu L, Han W (2023). Ferroptosis triggered by STAT1- IRF1-ACSL4 pathway was involved in radiation-induced intestinal injury. Redox biology.

[82] Zhou H, Zhou YL, Mao JA, Tang LF, Xu J, Wang ZX (2022). NCOA4-mediated ferritinophagy is involved in ionizing radiation-induced ferroptosis of intestinal epithelial cells. Redox biology.

[83] Dar HH, Epperly MW, Tyurin VA, Amoscato AA, Anthonymuthu TS, Souryavong AB (2022). P. aeruginosa augments irradiation injury via 15-lipoxygenase-catalyzed generation of 15-HpETE-PE and induction of theft-ferroptosis. JCI insight.

[84] Ji Q, Fu S, Zuo H, Huang Y, Chu L, Zhu Y (2022). ACSL4 is essential for radiation-induced intestinal injury by initiating ferroptosis. Cell death discovery.

[85] Chen Y, Wu M (2023). Exploration of molecular mechanism underlying protective effect of astragaloside IV against radiation-induced lung injury by suppressing ferroptosis. Archives of biochemistry and biophysics.

[86] Li L, Wu D, Deng S, Li J, Zhang F, Zou Y (2022). NVP-AUY922 alleviates radiation-induced lung injury via inhibition of autophagy-dependent ferroptosis. Cell death discovery.

[87] Li X, Zhuang X, Qiao T (2019). Role of ferroptosis in the process of acute radiation-induced lung injury in mice. Biochemical and biophysical research communications.

[88] Li X, Duan L, Yuan S, Zhuang X, Qiao T, He J (2019). Ferroptosis inhibitor alleviates Radiation-induced lung fibrosis (RILF) via down-regulation of TGF-β1. Journal of inflammation (London, England).

[89] Zhang X, Tian M, Li X, Zheng C, Wang A, Feng J (2021). Hematopoietic protection and mechanisms of ferrostatin-1 on hematopoietic acute radiation syndrome of mice. International journal of radiation biology.

[90] Zhang X, Xing X, Liu H, Feng J, Tian M, Chang S (2020). Ionizing radiation induces ferroptosis in granulocyte-macrophage hematopoietic progenitor cells of murine bone marrow. International journal of radiation biology.

[91] Gan F, Wang R, Lyu P, Li Y, Fu R, Du Y (2021). Plasma-Derived Exosomes Boost the Healing of Irradiated Wound by Regulating Cell Proliferation and Ferroptosis. Journal of biomedical nanotechnology.

[92] Ren C, Tan P, Gao L, Zeng Y, Hu S, Chen C (2023). Melatonin reduces radiation-induced ferroptosis in hippocampal neurons by activating the PKM2/NRF2/GPX4 signaling pathway. Progress in neuro-psychopharmacology & biological psychiatry.

[93] Wang X, Lu Y, Cheng X, Zhu X, Li D, Duan H (2023). Local Multiple-site Injections of a Plasmid Harboring Human MnSOD Mitigate Radiation-induced Skin Injury by Inhibiting Ferroptosis. Current drug delivery.

[94] Ding Y, Ma L, He L, Xu Q, Zhang Z, Zhang Z (2022). A strategy for attenuation of acute radiation-induced lung injury using crocetin from gardenia fruit. Biomedicine & pharmacotherapy = Biomedecine & pharmacotherapie.

[95] Xu Y, Tu W, Sun D, Chen X, Ge Y, Yao S (2021). Nrf2 alleviates radiation-induced rectal injury by inhibiting of necroptosis. Biochemical and biophysical research communications.

[96] Zhang M, Lan H, Peng S, Zhou W, Wang X, Jiang M (2023). MiR-223-3p attenuates radiation-induced inflammatory response and inhibits the activation of NLRP3 inflammasome in macrophages. International immunopharmacology.

[97] Gao J, Peng S, Shan X, Deng G, Shen L, Sun J (2019). Inhibition of AIM2 inflammasome-mediated pyroptosis by Andrographolide contributes to amelioration of radiation-induced lung inflammation and fibrosis. Cell death & disease.

[98] Yang C, Song C, Wang Y, Zhou W, Zheng W, Zhou H (2022). Re-Du-Ning injection ameliorates radiation-induced pneumonitis and fibrosis by inhibiting AIM2 inflammasome and epithelial-mesenchymal transition. Phytomedicine: international journal of phytotherapy and phytopharmacology.

[99] Chen Z, Wang B, Dong J, Li Y, Zhang S, Zeng X (2021). Gut Microbiota-Derived l-Histidine/Imidazole Propionate Axis Fights against the Radiation-Induced Cardiopulmonary Injury. International journal of molecular sciences.

[100] Yu H, Yao S, Zhou C, Fu F, Luo H, Du W (2021). Morroniside attenuates apoptosis and pyroptosis of chondrocytes and ameliorates osteoarthritic development by inhibiting NF-κB signaling. Journal of ethnopharmacology.

[101] Tan Y, Sun R, Liu L, Yang D, Xiang Q, Li L (2021). Tumor suppressor DRD2 facilitates M1 macrophages and restricts NF-κB signaling to trigger pyroptosis in breast cancer. Theranostics.

[102] Zhang F, Liu T, Huang HC, Zhao YY, He M, Yuan W (2022). Activation of pyroptosis and ferroptosis is involved in radiation-induced intestinal injury in mice. Biochemical and biophysical research communications.

[103] Liu T, Wu DM, Zhang F, Zhang T, He M, Zhao YY (2022). miR-142a-3p Enhances FlaA N/C Protection Against Radiation-Mediated Intestinal Injury by Modulating the IRAK1/NF-κB Signaling Pathway. International journal of radiation oncology, biology, physics.

[104] Guo J, Ding W, Cai S, Ren P, Chen F, Wang J (2023). Polydatin radiosensitizes lung cancer while preventing radiation injuries by modulating tumor-infiltrating B cells. Journal of cancer research and clinical oncology.

[105] Arroyo-Hernández M, Maldonado F, Lozano-Ruiz F, Muñoz-Montaño W, Nuñez-Baez M, Arrieta O (2021). Radiation-induced lung injury: current evidence. BMC pulmonary medicine.

[106] Loveless R, Bloomquist R, Teng Y (2021). Pyroptosis at the forefront of anticancer immunity. Journal of experimental & clinical cancer research: CR.

[107] Lhuillier C, Rudqvist NP, Elemento O, Formenti SC, Demaria S (2019). Radiation therapy and anti-tumor immunity: exposing immunogenic mutations to the immune system. Genome medicine.

[108] Zietman AL, Yom SS (2020). Radiation Therapy and the Immune System: A Scientific Revolution in the Making. International journal of radiation oncology, biology, physics.

[109] Cao W, Chen G, Wu L, Yu KN, Sun M, Yang M (2022). Ionizing Radiation Triggers the Antitumor Immunity by Inducing Gasdermin E-Mediated Pyroptosis in Tumor Cells. International journal of radiation oncology, biology, physics.

[110] Shi X, Yang Y, Zhang W, Wang J, Xiao D, Ren H (2022). FLASH X-ray spares intestinal crypts from pyroptosis initiated by cGAS-STING activation upon radioimmunotherapy. Proceedings of the National Academy of Sciences of the United States of America.

[111] Tan G, Lin C, Huang C, Chen B, Chen J, Shi Y (2022). Radiosensitivity of colorectal cancer and radiation-induced gut damages are regulated by gasdermin E. Cancer letters.

[112] Zhang Y, Li Z, Hong W, Hsu S, Wang B, Zeng Z (2023). STING-Dependent Sensing of Self-DNA Driving Pyroptosis Contributes to Radiation-Induced Lung Injury. International journal of radiation oncology, biology, physics.

[113] Jiang SJ, Xiao X, Li J, Mu Y (2023). Lycium barbarum polysaccharide-glycoprotein ameliorates ionizing radiation-induced epithelial injury by regulating oxidative stress and ferroptosis via the Nrf2 pathway. Free radical biology & medicine.

[114] Xie LW, Cai S, Zhao TS, Li M, Tian Y (2020). Green tea derivative (-)-epigallocatechin-3-gallate (EGCG) confers protection against ionizing radiation-induced intestinal epithelial cell death both in vitro and in vivo. Free radical biology & medicine.

[115] Tang LF, Ma X, Xie LW, Zhou H, Yu J, Wang ZX (2023). Perillaldehyde Mitigates Ionizing Radiation-Induced Intestinal Injury by Inhibiting Ferroptosis via the Nrf2 Signaling Pathway. Molecular nutrition & food research.

[116] Wu S, Tian C, Tu Z, Guo J, Xu F, Qin W (2023). Protective effect of total flavonoids of Engelhardia roxburghiana Wall. leaves against radiation-induced intestinal injury in mice and its mechanism. Journal of ethnopharmacology.

[117] Phinney DG, Pittenger MF (2017). Concise Review: MSC-Derived Exosomes for Cell-Free Therapy. Stem cells (Dayton, Ohio).

[118] Chen Z, Yao J, Zhang P, Wang P, Ni S, Liu T (2023). Minimized antibiotic-free plasmid vector for gene therapy utilizing a new toxin-antitoxin system. Metabolic engineering.

[119] Liu C, Liao W, Chen J, Yu K, Wu Y, Zhang S (2023). Cholesterol confers ferroptosis resistance onto myeloid-biased hematopoietic stem cells and prevents irradiation-induced myelosuppression. Redox biology.

[120] Wang X, Li W, Dong Y, Zhang Y, Huo Q, Lu L (2023). Ferrostatin-1 mitigates ionizing radiation-induced intestinal injuries by inhibiting apoptosis and ferroptosis: an in vitro and in vivo study. International journal of radiation biology.

[121] Zhang J, Li K, Zhang Q, Zhu Z, Huang G, Tian H (2021). Polycysteine as a new type of radio-protector ameliorated tissue injury through inhibiting ferroptosis in mice. Cell death & disease.

[122] Wang L, Wang A, Fu Q, Shi Z, Chen X, Wang Y (2022). Ferroptosis plays an important role in promoting ionizing radiation-induced intestinal injuries. Biochemical and biophysical research communications.

[123] Yin J, Hu N, Yi L, Zhao W, Cheng X, Li G (2021). Identification of Ferroptosis Biomarker in AHH-1 Lymphocytes Associated with Low Dose Radiation. Health physics.

[124] Thermozier S, Hou W, Zhang X, Shields D, Fisher R, Bayir H (2020). Anti-Ferroptosis Drug Enhances Total-Body Irradiation Mitigation by Drugs that Block Apoptosis and Necroptosis. Radiation research.

[125] Huang Z, Epperly M, Watkins SC, Greenberger JS, Kagan VE, Bayır H (2016). Necrostatin-1 rescues mice from lethal irradiation. Biochimica et biophysica acta.

[126] Wang Y, Wang Y, Pan J, Gan L, Xue J (2023). Ferroptosis, necroptosis, and pyroptosis in cancer: Crucial cell death types in radiotherapy and post-radiotherapy immune activation. Radiotherapy and oncology: journal of the European Society for Therapeutic Radiology and Oncology.

[127] NCT05340673.

[128] NCT01246973.

[129] NCT01932073.

[130] NCT02452463.

[131] NCT02452073.

[132] Dainiak N, Albanese J (2022). Medical management of acute radiation syndrome. Journal of radiological protection: official journal of the Society for Radiological Protection.

[133] Giridhar P, Mallick S, Rath GK, Julka PK (2015). Radiation induced lung injury: prediction, assessment and management. Asian Pacific journal of cancer prevention: APJCP.

[134] Smit SG, Heyns CF (2010). Management of radiation cystitis. Nature reviews Urology.

[135] NCT03613506.

[136] Moon JH, Jeong JK, Hong JM, Seol JW, Park SY (2019). Inhibition of Autophagy by Captopril Attenuates Prion Peptide-Mediated Neuronal Apoptosis via AMPK Activation. Molecular neurobiology.

[137] Meamar R, Dehghani L, Ghasemi M, Saadatnia M, Basiri K, Faradonbeh NA (2013). Enalapril protects endothelial cells against induced apoptosis in Alzheimer's disease. Journal of research in medical sciences: the official journal of Isfahan University of Medical Sciences.

[138] NCT01754909.

[139] Citrin DE (2017). Recent Developments in Radiotherapy. The New England journal of medicine.

[140] Chandra RA, Keane FK, Voncken FEM, Thomas CR Jr (2021). Contemporary radiotherapy: present and future. Lancet (London, England).

[141] Feng H, Liu Y, Gan Y, Li M, Liu R, Liang Z (2022). AdipoR1 Regulates Ionizing Radiation-Induced Ferroptosis in HCC cells through Nrf2/xCT Pathway. Oxidative medicine and cellular longevity.

[142] Van Opdenbosch N, Lamkanfi M (2019). Caspases in Cell Death, Inflammation, and Disease. Immunity.

[143] Tian W, Xu D, Han W, He H, Cai H, Chen H (2021). [Corrigendum] Cyclophilin D modulates cell death transition from early apoptosis to programmed necrosis induced by honokiol. International journal of oncology.

[144] Yang G, Lu C, Mei Z, Sun X, Han J, Qian J (2021). Association of Cancer Stem Cell Radio-Resistance Under Ultra-High Dose Rate FLASH Irradiation With Lysosome-Mediated Autophagy. Frontiers in cell and developmental biology.

[145] Su F, Duan J, Zhu J, Fu H, Zheng X, Ge C (2021). Long non-coding RNA nuclear paraspeckle assembly transcript 1 regulates ionizing radiation-induced pyroptosis via microRNA-448/gasdermin E in colorectal cancer cells. International journal of oncology.

[146] Li X, Chen J, Yuan S, Zhuang X, Qiao T (2022). Activation of the P62-Keap1-NRF2 Pathway Protects against Ferroptosis in Radiation-Induced Lung Injury. Oxidative medicine and cellular longevity.

[147] NCT05114226.

[148] NCT03557983.

[149] Zhang T, Wu DM, Luo PW, Liu T, Han R, Deng SH (2022). CircNEIL3 mediates pyroptosis to influence lung adenocarcinoma radiotherapy by upregulating PIF1 through miR-1184 inhibition. Cell death & disease.

[150] Smith AO, Ju W, Adzraku SY, Wenyi L, Yuting C, Qiao J (2021). Gamma Radiation Induce Inflammasome Signaling and Pyroptosis in Microvascular Endothelial Cells. Journal of inflammation research.

[151] Li C, Tian M, Gou Q, Jia YR, Su X (2019). Connexin43 Modulates X-Ray-Induced Pyroptosis in Human Umbilical Vein Endothelial Cells. Biomedical and environmental sciences: BES.

[152] Liu YG, Chen JK, Zhang ZT, Ma XJ, Chen YC, Du XM (2017). NLRP3 inflammasome activation mediates radiation-induced pyroptosis in bone marrow-derived macrophages. Cell death & disease.

[153] El-Benhawy SA, Elblehi SS, Hammoury SI, El-Soud AAA (2022). Studying ferroptosis and pyroptosis as new cell death mechanisms induced by ionizing radiation in Ehrlich solid tumor-bearing mice. Cancer treatment and research communications.

[154] Guo XW, Zhang H, Huang JQ, Wang SN, Lu Y, Cheng B (2021). PIEZO1 Ion Channel Mediates Ionizing Radiation-Induced Pulmonary Endothelial Cell Ferroptosis via Ca(2+)/Calpain/VE-Cadherin Signaling. Frontiers in molecular biosciences.

[155] Liu R, Liu L, Bian Y, Zhang S, Wang Y, Chen H (2021). The Dual Regulation Effects of ESR1/NEDD4L on SLC7A11 in Breast Cancer Under Ionizing Radiation. Frontiers in cell and developmental biology.

[156] Yuan Z, Liu T, Huo X, Wang H, Wang J, Xue L (2022). Glutamine Transporter SLC1A5 Regulates Ionizing Radiation-Derived Oxidative Damage and Ferroptosis. Oxidative medicine and cellular longevity.

[157] Zhao J, Tang M, Tang H, Wang M, Guan H, Tang L (2023). Sphingosine 1-phosphate alleviates radiation-induced ferroptosis in ovarian granulosa cells by upregulating glutathione peroxidase 4. Reproductive toxicology (Elmsford, NY).

