# Supplementary material for: The scheme, and regulative mechanism of pyroptosis, ferroptosis, and necroptosis in radiation injury
Source: Int J Biol Sci. 2024 Mar 3;20(5):1871–83. doi: 10.7150/ijbs.91112 (PMC10929204; doi:10.7150/ijbs.91112)
Supplement: Supplementary file 1 — Supplementary tables. [file ijbsv20p1871s1.pdf]

Supplementary Table 1 Dose for pyroptosis-, ferroptosis- and necroptosis-related radiation injury

Affections	Disease	Cell line/animal	Radiation regimen (dose/dose rate)	Reference
Pyroptosis	Sepsis in radiotherapy	EA. hy926 cells; cGas-/-, Caspase-11-/- mice, GSDMD/-, Asc-/- mice	14 Gy; 2.5 Gy/min	[72]
Pyroptosis	RI-lung injury	bone marrow cells	16 Gy	[112]
Pyroptosis	RI-lung injury (inflammation, fibrosis)	Male Sprague-Dawley (SD) rats, (210-230 g)	20 Gy (3 Gy/min)	[73]
Pyroptosis	RI-pneumonitis and fibrosis	C57BL/6 mice (female, 8-week-old)	18 Gy (X-ray)	[98]
Pyroptosis	RI-pneumonitis and fibrosis	C57BL/6 mice (female, 6-8 weeks old, 18-22 g)	Cell: 8Gy (X-ray); 297.43 cGy/min; Animal: 18Gy (X-ray); 2 Gy/min	[97]
Pyroptosis	RI-liver pyroptosis	ICR mice	0.25-1 Gy	[74]
Pyroptosis	RI-intestinal Injury	C57BL/6J mice	10 Gy; 2 Gy/min	[77]
Pyroptosis	RI-intestinal Injury	C57BL/6 mice (female, 6-8 weeks)	9 Gy	[78]
Pyroptosis	RI-intestinal Injury	C57BL/6 mice (male)	4 Gy	[79]
Pyroptosis	RI-cardiopulmonary Injury	BEAS-2B; C57BL/6J mice (6-8-week-old-male, around 20 g)	Cell: 4-6 Gy; Animal: 15 Gy (γ-ray); 0.8 Gy/min	[99]
Pyroptosis	RI-brain injury	BV-2 microglia cells; BALB/c mice	Cell: 10 Gy; 3MeV/min; Animal: 15 Gy; 3 Gy/min	[75]
Pyroptosis	RI-salivary gland damage	HUVECs; C57BL/6 mice (female, 6-week-old, 18-24 g)	Cell: 8 Gy; 4 Gy/min; Animal: 15 Gy; 4 Gy/min	[76]
Pyroptosis, apoptosis	RI-intestinal Injury	BALB/c mice (female)	10 Gy; 2 Gy/min	[80]
Pyroptosis, ferroptosis	RI-intestinal Injury	C57BL/6 mice	5, 10 Gy; 2 Gy/min	[102]
pyroptosis	RI-intestinal injury	HIEC-6, HT29, sw620, LS174T; C57BL/6 mice (male, 6-8 weeks, 20-22 g)	Cell: 4Gy, 8Gy; Animal: 13Gy (0.99 Gy/min)	[116]
Pyroptosis	Double-edge	CT26; BALB/c mice (6-8 weeks, female); Wild-type (WT) and Gsdme-/- mice (C57BL/6 strain)	8 Gy; 2 Gy/fraction at day 7, 14, 21, 28	[111]

Pyroptosis	NA	Microvascular brain endothelial cells (ECs)	NA Gy (γ-ray, 1.8 Gy/min)	[150]
Pyroptosis	NA	HUVEC	10 Gy (X-ray)	[151]
Pyroptosis	NA	Macrophages	10 Gy (60Co)	[152]
Pyroptosis, ferroptosis	NA	Ehrlich solid tumor-bearing mice	6 Gy, 2*6 Gy, 3*4 Gy, 4*2 Gy (X-ray); 200 Mu/min	[153]
Ferroptosis	Double-edge; Radiation resistance; RI-intestinal injuries	IEC-6	9 Gy (1.0 Gy/min)	[120]
Ferroptosis	RI-myelosuppression	Mouse hematopoietic stem cells; C57BL/6 mice, ApoE-/-, Ldlr-/-	Animal: 5 Gy, 7.5 Gy (92.8-95.5 cGy/min)	[119]
Ferroptosis	RI-epithelial injury	HaCaT; Sprague-Dawley rats (160 ± 20 g)	Cell: 12 Gy (6 Gy/min); Animal: 10 Gy (4 Gy/min)	[113]
Ferroptosis	RI-hippocampal neuron injury	HT-22; C57BL/6 J mice (male)	Cell: 5 Gy; Animal: 8 Gy	[92]
Ferroptosis	RI-skin injury	HaCaT; Sprague-Dawley (SD) rats (female, 200 ± 10g)	Cell: 20 Gy; Animal: 40 Gy	[93]
Ferroptosis	Healing of Irradiated Wound	NIH/3T3 mouse embryonic fibroblasts; SD rats (male, 10-11 weeks old, 250-300 g)	Cell: 4 Gy (60Co, γ-ray, 3 Gy/min); Animal: 6 Gy	[91]
Ferroptosis	hematopoietic acute radiation syndrome	ICR mice (male, 8-10 weeks old)	Cell: 8 Gy; Animal: 8, 10 Gy; 2 Gy/min	[89]
Ferroptosis	RI bleeding	granulocyte-macrophage hematopoietic progenitor cells; ICR mice (male, 8-10 weeks)	Cell: 4-8 Gy; Animal: 10 Gy; 2 Gy/min	[90]
Ferroptosis, apoptosis	RI-intestinal injury	C57BL/6J; HIEC-6, intestinal crypt organoids	Cell: 6 Gy; Animal: 13 Gy	[115]
Ferroptosis	RI-intestinal injury	Male C57BL/6J mice aged 6-8 weeks (21-25 g); FHs74Int, intestinal organoids	Cell: 0.189 Gy/min; Animal: 11, 14, 15, 17 Gy (6 Gy/min)	[81]
Ferroptosis	RI-intestinal injury	HIEC; C57BL/6J mice (4-8-week-old male)	Cell: 6 Gy (X-ray, 1.1 Gy/min); Animal: 14 Gy (X-ray, 1.1 Gy/min)	[82]

Ferroptosis	RI-intestinal injury	C57BL/6 mice (female, 6-8 weeks, 25 g)	14 Gy (400 Mu/min)	[84]
Ferroptosis	RI-lung fibrosis	C57BL/6 mice (4-6 weeks old, female)	15 Gy (X-ray, 2 Gy/min)	[88]
Ferroptosis	RI-lung injury	A549; C57BL/6 mice (female, 5-7 weeks old, 19-23 g)	Cell and Animal: 10 Gy (2 Gy/min)	[85]
Ferroptosis	RI-lung injury	HULEC-5a; C57BL/6 mice	15 Gy	[154]
Ferroptosis	RI-lung injury	BEAS-2B; C57BL/6 mice	Cell: 15 Gy; Animal: 10 Gy	[86]
Ferroptosis	RI-lung injury	C57BL/6 mice (female, 20 ± 2 g)	15Gy (6MV, X-ray)	[87]
Ferroptosis	RI-multi-organ injury	IEC6; male C57BL/6 mice, BALB/c-Nude mice	Cell: 8 Gy or 10 Gy (γ-ray, 0.99 Gy/min); Animal: WBI: 4Gy, 7.2 Gy, 7.5 Gy, 10 Gy, 12.5 Gy (0.99 Gy/min, γ ray); ABI: 15 Gy, 18 Gy (γ ray, 0.99 Gy/min); lung fibrosis: 17 Gy (X-ray, 0.99 Gy/min, 3.23 Gy/min)	[121]
Ferroptosis, apoptosis	RI-intestinal injuries	HIEC; C57 BL/6 J mice (6-8 weeks, male)	Cell: 4 Gy (X-ray, 1.15 Gy/min); Animal: 9 Gy (60Co, γ-ray, 2 Gy/min)	[114]
Ferroptosis, apoptosis	RI-intestinal injuries	Balb/c mice (male, 20 ± 2 g)	2 Gy (60Co, γ-ray, 1 Gy/min)	[122]
Ferroptosis, apoptosis	RI-intestinal injury	Caco2 (HTB-37), HIEC6 (CRL-3266); C57BL/6J mice (7-8 weeks old, female)	9.25 Gy	[83]
Ferroptosis	RI-lung injury	A549	10 Gy (X-ray,2 Gy/min)	[146]
Ferroptosis	NA	AHH-1 lymphocytes	2.4, 4.8, 7.2, 28.8 mGy (7, 14, 26, 34, 43μ Gy/h, γ-ray)	[123]
Ferroptosis	NA	ER-positive (MCF-7 and ZR-75-1) and ER-negative (MDA-MB-231)	8 Gy (X-ray)	[155]
Ferroptosis	NA	HepG2, SMMC-7721, Huh7, Huh6, HT1080; HepG2-IRR (2, 4, 6, 8 Gy, 6 Gy/min)	Cell: 10 Gy (6 Gy/min)	[156]
Ferroptosis	NA	KGN	2 Gy	[157]
Necroptosis	RI-rectal injury	HIEC; C57BL/6mice (female, 5-week-old, 20 g)	Cell: 0, 1, 2, 4, 6, 8 Gy (3.6 Gy/min, X-ray); Animal: 25 Gy (X-ray, 3.6 Gy/min)	[95]
Necroptosis	RI-injury	Mouse embryonic Cyt c-/- cells, Cyt c+/+ cells; C57BL/6NTac mice (female)	9.25 Gy, 9.5 Gy (80 cGy/min)	[125]

Necroptosis	RI-injury	C57BL/6NTac mice (female, 30-33 g); Bone marrow stromal cell lines, hematopoietic progenitor cell lines	Cell: 0-8 Gy; Animal: 9.25 Gy (TBI, 343 cGy/min)	[124]
Necroptosis	RI-lung injury	C57BL/6 mice	15 Gy (X-ray, 200 cGy/min)	[94]

RI: radiation-induced; RT: radiotherapy; NA: not applicable.

Supplementary Table 2 The agents and regulative mechanism for pyroptosis-, ferroptosis- and necroptosis-related radiation injury

Affections	Disease	Cell line/animal	Drugs	Indicators	PMID
Pyroptosis	RI-lung injury (inflammation, fibrosis)	Male Sprague-Dawley (SD) rats, (210-230 g)	ACT001 (50, 100, 200 mg/kg)	ACT001→IL-6, TNF-α, IL-1β, NLRP3, ASC, GSDMD↓, LDH→pyroptosis↓→IR-lung injury--EMT (TGF-β1, E-cadherin, α-smooth muscle actin (α-SMA), vimentin)↓	[73]
Pyroptosis	RI-cardiopulmonary injury	BEAS-2B; C57BL/6J mice (6-8-week-old-male, around 20 g)	gut microbiota-derived I-Histidine; Cell: imidazole propionate (ImP) (8 μL/mL)	IR+gut microbiota-derived I-Histidine: radiation toxicity in lung and heart↓; I-Histidine→rely on gut microbiota configuration and metabolome changes, assimilation of imidazole propionate (maybe gram-positive bacteria)→radioprotection<br>I-Histidine and ImP→proliferation<br>IR: GSDMD, TNF-α, NF-κB↑<br>IR+ImP→pyroptosis (GSDMD, caspase-1, caspase-4, caspase-5)↓, F-actin↑	[99]
Pyroptosis	RI-brain injury	BV-2 microglia cells; BALB/c mice	Mesenchymal stem cells (MSCs)	IR+MSCs→NLRP3↓, caspase-1↓, ROS↓→caspase-1 dependent pyroptosis↓	[75]
Pyroptosis	RI-lung injury	Bone marrow cells	Pulmozyme	RT→dsDNA→cGAS-STING-NLRP3→IL-1β, IL-18, GSDMD-N↑→pyroptosis↑→IR-lung injury↑; Pulmozyme→dsDNA↓→IR-lung injury↓	[112]
Pyroptosis	RI-intestinal Injury	C57BL/6J mice	Micheliolide: Cell: 10μM; Animal: 50mg/kg	mucosal injury, necrosis, loss of tissue structure, edema, and inflammatory cell infiltration↓; IR+Micheliolide→survival↑, body weigh↑, IL-1β, IL-18, TGF-β1, TNF-α, IFN-γ ↓, caspase-1, cleaved caspase-1, GSDMD-N ↓, autophagosomes and autolysosomes, LC3, p62, beclin 1↑→NLRP3 inflammasome-dependent pyropyosis↓	[77]
Pyroptosis	RI-intestinal Injury	C57BL/6 mice (female, 6–8 weeks)	5-AED	IR+5-AED: caspase-1 p10↓+GSDMD-N↓+NF-κB↑, pyroptosis ↓; AIM2, ASC=↓interaction	[78]
Pyroptosis	RI-liver pyroptosis	ICR mice	rMnSOD	rMnSOD+IR→caspase1↓, SMPD1↑, SMPD2↓ (limited contral in SMPD1), aSMase↓, nSMase1↓→IR-liver pyroptosis↓	[74]
Pyroptosis	RI-salivary gland damage	HUVECs; C57BL/6 mice (female, 6-week-old, 18–24 g)	NaNO3 (0.1, 0.5, 2, 5 mmol/L)	IR+nitrate (NaNO3) →complex I ↑, ROS↓, IL-1β =/↓, GSDMD↓, GSDMD-NT↓, NLPR3↓, ASC↓, IL-18↓→acinar cells and microvascular endothelial cells↑+NLPR3-mediated pyroptosis↓	[76]

Pyroptosis, apoptosis	RI-intestinal Injury	BALB/c mice (female)	Flagellin A N/C (FlaAN/C): 1mg/kg	IR+FlaAN/C→ROS, NLRP3, caspase-1, IL-1β, IL-18, IL-8, IL-6, TNF-α, MCP-1↓→pyroptosis+apoptosis↓→villi↑, cellular infiltration and hemorrhage areas↓	[80]
Pyroptosis, apoptosis	RI-intestinal Injury	C57BL/6 (male)	p-Coumaric acid (CA)	IR+CA→caspase-1, NLRP3, AIM2↓, Occludin, ZO-1, Claudin-5↑→intestinal injury↓, apoptosis↓, antioxidant indexes↑, inflammation response↓	[79]
Pyroptosis, ferroptosis	RI-intestinal Injury	C57BL/6	PDTC	IR+VX-765→IL-18, IL-1β, caspase 1↓→pyroptosis↓→injury↓; IR+Fer-1→GPX4↓, iron ↑, 4-hydroxynonenal (4-HNE) ↑, MDA↑, GSH↓→lipid peroxidation↓→ferroptosis↓→injury↓; PDTC→NF-κB↓→injury↓ above indicators occur	[102]
Pyroptosis	Sepsis in radiotherapy	EA.hy926 cells; cGas-/-, caspase-11-/- mice, GSDMD/- , Asc-/- mice	NA	deficiency of cGAS→survival↑, radiation-promoted sepsis↓, caspase-11↓, IFNβ, IL-1α, IL-1β↓, permeability of alveolar capillaries↓, edema↓; cGAS↓→caspase-11↓→pyroptosis↓→radiation-promoted sepsis↓	[72]
Ferroptosis	Double-edge; RI-intestinal injury	HIEC-6, HT29, sw620, LS174T; C57BL/6 mice (male, 6-8 weeks, 20-22 g)	Engelhardia roxburghiana Wall. Leaves	Engelhardia roxburghiana Wall. leaves →NRF2↑, HO-1, NQO1↑→ SOD, GSH, IL-6, TNF-α↓→IR-intestinal injury↓, radiosensitivity↑	[116]
Ferroptosis	RI-epithelial injury	HaCaT; Sprague-Dawley rats (160 ± 20 g)	Lycium barbarum polysaccharide-glycoprotein	Lycium barbarum polysaccharide-glycoprotein (LBP)→NRF2↑→HO-1, NQO1, SLC7A11, FTH1↑→ferroptosis↓→IR-epithelial injury↓	[113]
Ferroptosis	RI-lung injury	A549; C57BL/6 mice (female, 5-7 weeks old, 19-23 g)	Astragaloside IV (20 or 40 mg/kg)	RT→Fe2+, LDH, MDA, IL-6, TNF-α, IL-1β↑, NRF2, HO1, NQO1, GSH ↓→ferroptosis↑→IR-lung injury↑; Astragaloside IV→ferroptosis↓→IR-lung injury↑	[85]
Ferroptosis, apoptosis	RI-intestinal injury	C57BL/6J; HIEC-6, intestinal crypt organoids	Perillaldehyde	Perillaldehyde→NRF2, GPX4, GSH↑, ROS, PTGS2↓→ferroptosis, apoptosis, DNA damage↓→proliferation, differentiation→intestinal permeability, barrier damage↓→survival↑	[115]
Ferroptosis, apoptosis	RI-intestinal injury	HIEC; C57 BL/6 J mice (6-8-week-old male)	Green tea derivative (-)-epigallocatechin-3-gallate (EGCG) (Cell: 2μMl, Animal: 12.5,25mg/kg)	EGCG: NRF2↑, translocation of NRF2 from the cytoplasm to the nucleus↑→ SLC7A11, HO-1, GPX4↑→ ROS↓→DNA damage, apoptosis, γH2AX foci, ferroptosis↓, Lgr5+ intestinal stem cells (ISCs), ISCs Ki67+ cells↑→intestinal mucosal injury↓, survival time↑	[114]

Ferroptosis, apoptosis	RI-intestinal injury	Caco2 (HTB-37), HIEC6 (CRL-3266); C57BL/6J mice (7 8 weeks old, female)	PAO1	pseudomonas aeruginosa (PAO1)→antiferroptotic system, GSH/GPX4↓, 15-HpETE-PE↑→oxidized phosphatidylethanolamine (PEox)↑→ferroptosis↑, survival↓	[83]
Ferroptosis	RI-skin injury	HaCaT; Sprague-Dawley (SD) rats (female, 200 ± 10g)	Plasmid encoding human MnSOD	Plasmid encoding human MnSOD→ ROS, ACSL4↓, GPX4, SLC7A11↑→ferroptosis↓→SOD↑→IR-skin injury↓	[93]
Ferroptosis	Healing of irradiated wound	NIH/3T3 mouse embryonic fibroblasts; SD rats (male, 10-11 weeks old, 250–300 g)	RPExos (from healthy rats, Cell: 400 g/ml exosomes); Ferrostatin-1	RPExos↑→GPX4, NRF2, FTH1↑, Fe2+↓→ferroptosis ↓→healing↑; RPExos↑→proliferation, migration, cell cycle progression (G0/G1↓, S↑), cell survival↑, cell proliferation and radioresistance-related genes↑, ferroptosis ↓	[91]
Ferroptosis	RI-hippocampal neuron injury	HT-22; C57BL/6 J mice (male)	Melatonin (10mg/kg in animal)	Melatonin→PKM2 bing to NRF2 thus entering the nucleus↑→GPX4↑→ferroptosis↓→IR-hippocampal neuron injury↓	[92]
Ferroptosis	RI-myelosuppression	Mouse hematopoietic stem cells; C57BL/6 mice, ApoE-/-, Ldlr-/-	Cholesterol c	Cholesterol→SLC38A9–mTOR →SLC7A11/GPX4 ↑ferritinophagy↓→ferroptosis resistance of myeloid-biased hematopoietic stem cells↑→IR-myelosuppression↓	[119]
Ferroptosis	RI-intestinal injury	Male C57BL/6J mice aged 6-8 weeks (21-25 g); FHs74Int, intestinal organoids	Ferrostatin-1 (5mg/kg/d)	RT→STAT1-IRF1-ACSL4→GPX4↓, SLC3A2↓→ferroptosis↑→IR-intestinal injury↑; Ferrostatin-1→ferroptosis, IR-intestinal injury↓→survival↑	[81]
Ferroptosis	RI-intestinal injury	HIEC; C57BL/6J mice (4-8 weeks, male)	si NCOA4; DFO (100mg/kg)	IR→NCOA4→ferritinophagy↑→iron↑→MFRN2↑, TRF1↑, FPN1, FTL, FTH↓→extensive mitochondrial fragmentation and peri-nuclear aggregation↑→iron to nuclear↑, ROS↑→ferroptosis↑; iron-deficient diet→IR-induced intestinal injury↓	[82]
Ferroptosis	Double-edge; Radiation resistance; RI-intestinal injuries	IEC-6	Ferrostatin-1	Ferrostatin-1 → GPX4↓→ferroptosis↓, gut-microbe balance restore→IR-intestinal injuries↓, radioresistance↓	[120]
Ferroptosis	RI-lung fibrosis	C57BL/6 mice (4-6 weeks old, female)	Liproxstatin-1 (10 mg/kg)	liproxstatin-1→NRF2, HO1, NQO1↑→TGF-β1↓→RILF↓; IR: GPX4↓→ferroptosis↑; liproxstatin-1→GPX4↑, ROS, HYP↓→ferroptosis↓	[88]
Ferroptosis	RI-lung injury	BEAS-2B; C57BL/6 mice	NVP-AUY922 (Cell: 10nM, Animal: 10mg/kg)	NVP-AUY922→HSP90-mediated CMA pathway↓→lysosomal degradation of GPX4↓→ferroptosis↓→IR-induced lung injuries, inflammatory cell infiltration,	[86]

				proinflammatory cytokine release, and lung epithelial BEAS-2B cell damage (TNF-α, IL-6, IL-1β↓) ↓	
Ferroptosis	RI-lung injury	C57BL/6 mice (female, 20 ± 2 g)	Liproxstatin-1 (10mg/kg once daily for 30 days)	liproxstatin-1: GPX4↑, ROS↓→ TNF-α, IL-6, IL-10, TGF-β1↓→ferroptosis↓→ structure damage and hemorrhage↓	[87]
Ferroptosis	RI-multi-organ injury	IEC6; male C57BL/6 mice, BALB/c-Nude mice	Compound 5 (1/2 maximum tolerance dose, 517mg/kg)	Compound 5→highest binding capability, survival rate: 80%, TBI-induced hematopoietic system injury↓, IR-induced lung injury↓; Compound 5→NOX1↓→total ROS and lipid ROS↓ (GSH, GPX4↑) →ferroptosis↓→TBI-induced gastrointestinal (GI) syndrome↓	[121]
Ferroptosis	RI-lung injury	A549	NA	IR: ROS↑→ferroptosis↑; P62, NRF2↑→GSH↓, MDA↑, mitochondrial membrane↓, GPX4↓, ACSL4↑, HO1, FTH1, NQO1↑→ferroptosis↑, IR-induced lung injury↑	[146]
Ferroptosis	RI-intestinal injury	C57BL/6 (female mice, 6-8 weeks, 25 g)	NA	IR: intestinal bacteria and fungi↑→ACSL4↑→ferroptosis↑→IR-induced intestinal injury↑	[84]
Ferroptosis	RI-lung injury	HULEC-5a; C57BL/6 mice	NA	IR: PIEZO1↑→calcium influx, calpain activity↑→degradation of VE-cadherin↑→ROS, lipid peroxidation, mitochondria shrinkage↑, GPX4, SLC7A11 ↓→ferroptosis↑→IR-lung injury↑	[154]
Necroptosis	RI-injury	C57BL/6NTac mice (female, 30-33 g); Bone marrow stromal cell lines, hematopoietic progenitor cell lines	JP4–039 (20 mg/kg), necrostatin-1 (1.65 mg/kg) and/or lipoxygenase-15 inhibitor (baicalein) (50 mg/kg) in single-, dual-or three-drug regimens	Drug→survival↑	[124]
Necroptosis	RI-lung injury	C57BL/6 mice	Crocetin: 100, 200 mg/Kg	Crocetin →Tnfrsf10b↓→necroptosis↓→IR-lung injury↓	[94]
Necroptosis	RI-rectal injury	HIEC; C57BL/6mice (female, 5-week-old, 20 g)	NA	IR→necroptosis↑; NRF2→necroptosis↓→IR-injury↓	[95]

Necroptosis	RI-injury	Mouse embryonic Cyt c-/- cells, Cyt c+/+ cells; C57BL/6NTac mice (female)	Necrostatin-1	Necrostatin-1→RIPK1, p-RIPK3↓→necroptosis↓→survival after lethal irradiation↑	[125]

RI: radiation-induced; RT: radiotherapy; IR: ionizing radiation; NA: not applicable.

Supplementary Table 3 Immune regulation in pyroptosis- and ferroptosis-related radiation injury

Affections	Disease	Immune cell	Cell line/animal	Drugs	Indicators	Reference
Pyroptosis	RI-lung injury	Macrophages	ANA-1, MH-S, C57BL/6 mice (female, 6-8 weeks, weight: 18-22 g)	MiR-223-3p	IR→caspase 1-p10, GSDMD-N, NLRP3, IL-18, IL-1β, TNF-α, IL-1α, CCL-2, LDH↑→IR-lung injury↑; MiR-223-3p→NLRP3↓→macrophages pyroptosis↓→inflammatory response, IR-lung injury↓	[96]
Pyroptosis	RI-pneumonitis and fibrosis	T lymphocytes, macrophages, neutrophils	C57BL/6 mice (female, 8-week-old)	Re-Du-Ning (RDN)	IR+RDN: IL-1β, IL-6, TNF-α, AIM2-mediated pyroptosis↓; RDN→EMT (α-SMA, E-cadheri, CDH1, CDH2, VIM, and ACTA2)↓→fibrosis↓; RDN→p-PI3K↓→p-AKT↓→fibrosis↓; RDN→AIM2 inflammasome↓→pyroptosis↓; RDN+4, 8, and 20 weeks after radiation: edema, hyperemia, inflammatory cell infiltration↓; immune cell infiltration: T lymphocytes, macrophages, neutrophils, BALF↓; IL-1β, IL-6, TNF-α↓	[98]
Pyroptosis	RI-pneumonitis and fibrosis	Macrophages	C57BL/6 mice (female, 6-8 weeks old, 18-22 g)	Andrographolide (5, 10, 20 mg/kg)	IR+Andrographolide→bone marrow-derived macrophages (BMDMs) AIM2 translocating into the nucleus↓→pyroptosis↓; IR-induced lung tissue damage, inflammatory cell infiltration, and pro-inflammatory cytokine release, EMT, progressive fibrosis, caspase-1, IL-1β, pyroptosis↓, NF-κB, MAPK↓ in an ROS-independent manner	[97]
Pyroptosis	Double-edge	NK cells	CT26; BALB/c mice (6-8 weeks, female); Wild-type (WT) and Gsdme-/- mice (C57BL/6 strain)	GSDME KO	GSDME: IECs > CRC; GSDME→caspase-3↑→pyroptosis↑→NK cells activation and recruition→radioresistance and radiation injury (intestine, stomach, liver and pancreas) ↓	[111]
Pyroptosis	Anti-tumor Immunity	T lymphocytes	PC-9, SF126, U251, HepG2, SF268, Huh-7, HCCLM3, MHCC-97L, T47D, THP1, 4T1, U87MG, MCF-7, MDA-MB-231; H1299, Calu-1, A172, BT549, H460,	Cisplatin/etoposide/decitabine/azacytidine	cisplatin/etoposide/decitabine/azacytidine + IR: →pyroptosis↑; IR→caspase-9↑→caspase-3↑→GSDME↑→pyroptosis↑→cytotoxic T lymphocytes↑+ cytokines↑	[109]

			H226, MDA-MB-468, Hep3B; Balb/c mice			
Pyroptosis	RI-intestinal Injury	CD8+ T cells	C57BL/6 mice	anti-PD-L1	FLASH-X ray vs. conventional RT in PD-L1 KO mouse: CD8+ T cells, pyroptosis, GSDME-NT↓	[110]
Ferroptosis	Hematopoietic acute radiation syndrome	Lymphocytes, monocytes	ICR mice (male, 8-10 weeks old)	Ferrostatin-1 (2 mg/kg)	Ferrostatin-1: hemosiderin and liable iron pool↓, ASCL4 and GPX4↑, TNF-a, IL-6, IL-1β→ferroptosis of BMMCs↓→red and white blood cells, lymphocytes, monocytes↑→survival rate↑	[89]
Ferroptosis	RI-bleeding	Lymphocytes	Granulocyte-macrophage hematopoietic progenitor cells; ICR mice (male, 8-10 weeks)	LDN 193189 (3 mg/kg); Ferrostatin-1 (2 mg/kg); DFO (50/140/210 mg/kg)	IR: iron content in bone marrow↑, iron metabolism (Ferroportin-1↑, iron regulatory protein 1 and 2↓) →white blood cells and lymphocytes counts↓→ferroptosis↑→GM-HPC counts↓; IR+LDN: white blood cells and lymphocytes counts↑, iron content, hepcidin↓→survival↑	[90]
Ferroptosis, apoptosis	RI-intestinal injuries	Lymphocytes	Balb/c mice (male, 20 ± 2 g)	Liproxstatin-1 (10mg/kg once daily for 30 days)	liproxstatin-1→LPCAT3, ALOX15↑→mitochondrial damage↓, MDA↓→ferroptosis↓, intraepithelial lymphocytes↑, IL-17↑, TGF-β, IFN-γ↓	[122]
Ferroptosis	NA	Lymphocytes	AHH-1 lymphocytes	NA	0-4.8 mGy: TFRC1, SLC3A2, SLC39A8, FTH1, ACSL4, GPX4↓; 4.8-28.8 mGy: TFRC1, SLC3A2, SLC39A8, FTH1, ACSL4, GPX4↑	[123]

RI: radiation-induced; RT: radiotherapy; IR: ionizing radiation; NA: not applicable.
